# User Engagement With Smartphone Apps and Cardiovascular Disease Risk Factor Outcomes: Systematic Review

**DOI:** 10.2196/18834

**Published:** 2021-02-03

**Authors:** Erin M Spaulding, Francoise A Marvel, Rebecca J Piasecki, Seth S Martin, Jerilyn K Allen

**Affiliations:** 1 Johns Hopkins University School of Nursing Baltimore, MD United States; 2 The Welch Center for Prevention, Epidemiology and Clinical Research Johns Hopkins University Bloomberg School of Public Health Baltimore, MD United States; 3 Digital Health Innovation Laboratory, Ciccarone Center for the Prevention of Cardiovascular Disease Division of Cardiology, Department of Medicine Johns Hopkins University School of Medicine Baltimore, MD United States; 4 Johns Hopkins University School of Medicine Baltimore, MD United States; 5 Johns Hopkins University Whiting School of Engineering Baltimore, MD United States; 6 Johns Hopkins University Bloomberg School of Public Health Baltimore, MD United States

**Keywords:** mHealth, smartphone, mobile phone, engagement, cardiovascular disease, health behaviors, risk factors

## Abstract

**Background:**

The use of mobile health (mHealth) interventions, including smartphone apps, for the prevention of cardiovascular disease (CVD) has demonstrated mixed results for obesity, hypercholesterolemia, diabetes, and hypertension management. A major factor attributing to the variation in mHealth study results may be mHealth user engagement.

**Objective:**

This systematic review aims to determine if user engagement with smartphone apps for the prevention and management of CVD is associated with improved CVD health behavior change and risk factor outcomes.

**Methods:**

We conducted a comprehensive search of PubMed, CINAHL, and Embase databases from 2007 to 2020. Studies were eligible if they assessed whether user engagement with a smartphone app used by an individual to manage his or her CVD risk factors was associated with the CVD health behavior change or risk factor outcomes. For eligible studies, data were extracted on study and sample characteristics, intervention description, app user engagement measures, and the relationship between app user engagement and the CVD risk factor outcomes. App user engagement was operationalized as general usage (eg, number of log-ins or usage days per week) or self-monitoring within the app (eg, total number of entries made in the app). The quality of the studies was assessed.

**Results:**

Of the 24 included studies, 17 used a randomized controlled trial design, 4 used a retrospective analysis, and 3 used a single-arm pre- and posttest design. Sample sizes ranged from 55 to 324,649 adults, with 19 studies recruiting participants from a community setting. Most of the studies assessed weight loss interventions, with 6 addressing additional CVD risk factors, including diabetes, sleep, stress, and alcohol consumption. Most of the studies that assessed the relationship between user engagement and reduction in weight (9/13, 69%), BMI (3/4, 75%), body fat percentage (1/2, 50%), waist circumference (2/3, 67%), and hemoglobin A_1c_ (3/5, 60%) found statistically significant results, indicating that greater app user engagement was associated with better outcomes. Of 5 studies, 3 (60%) found a statistically significant relationship between higher user engagement and an increase in objectively measured physical activity. The studies assessing the relationship between user engagement and dietary and diabetes self-care behaviors, blood pressure, and lipid panel components did not find statistically significant results.

**Conclusions:**

Increased app user engagement for prevention and management of CVD may be associated with improved weight and BMI; however, only a few studies assessed other outcomes, limiting the evidence beyond this. Additional studies are needed to assess user engagement with smartphone apps targeting other important CVD risk factors, including dietary behaviors, hypercholesterolemia, diabetes, and hypertension. Further research is needed to assess mHealth user engagement in both inpatient and outpatient settings to determine the effect of integrating mHealth interventions into the existing clinical workflow and on CVD outcomes.

## Introduction

### Background

Heart disease remains the leading cause of death in the United States [1]. In 2011, the American Heart Association (AHA) set a strategic impact goal of decreasing deaths from cardiovascular diseases (CVDs) and stroke by 20% by 2020; thus, efforts have been made to improve health behavior and reduce the prevalence of risk factors for heart disease, including smoking, overweight and obesity, physical inactivity, poor nutrition, diabetes mellitus, hypercholesterolemia, and hypertension [1]. According to the 2015 National Health Interview Survey, 79% of US adults reported not achieving adequate physical activity (PA) [1]. According to the 2014 National Health and Nutrition Examination Survey, 46% of US adults have hypertension (based on the 2017 American College of Cardiology and AHA guidelines), 40% of US adults have moderately elevated or high total cholesterol, and 38% of US adults are obese [1]. Although still important risk factors for CVD, the prevalence of smoking and diagnosed diabetes is 15% and 9%, respectively, among US adults, which are lower than the other risk factors [1]. However, the prevalence of diabetes is increasing, whereas the prevalence of smoking among adults is decreasing [1].

Mobile health (mHealth), “the use of mobile computing and communication technologies, [such as smartphone applications]...for health services and information,” is an innovative approach that could be a potentially effective means of involving individuals in health promotion and CVD management [2]. Although the prevalence of smartphone ownership among adults in the United States is high [3-6], including 73% smartphone ownership among individuals with CVD risk [7], a state-of-the-science article demonstrated that there are conflicting findings about the effectiveness of mHealth interventions for CVD prevention in improving CVD health behavior change and risk factor outcomes, such as weight management, PA, hypertension management, diabetes management, and lipid control [2]. One potential but important cause of conflicting results may be differential user engagement with the interventions. However, directly assessing the relationship between user engagement with smartphone apps and CVD health behavior change and risk factor outcomes was not a goal of that review.

Engagement with (smartphone app) interventions is considered a precondition for effectiveness and is of particular concern for behavior change interventions [8]. Although the field of user engagement with health interventions is in the early stages, there is work that has been conducted to reach a consensus on how best to conceptualize and operationalize user engagement with these interventions [8-10]. For this systematic review, user engagement with smartphone apps is conceptually defined as the “emotional, cognitive, and behavioral experience of a user with a [smartphone application] that exists, at any point in time and over time, [to varying degrees]” [11]. User engagement is a dynamic process that likely coincides with behavior change to ultimately improve health outcomes [8]. Yardley et al [8] proposed one potential model, including 4 phases, of this process. In phase 1, individuals would engage with the smartphone app and prepare for behavior change [8,12]. In phase 2, individuals would partake in behavior change, mediated by sustained user engagement [8,12]. In phase 3, individuals would continue to partake in behavior change but may disengage from the smartphone app if no longer needed to sustain behavior change [8,12]. Finally, in phase 4, individuals may re-engage with the smartphone app if there is a lapse in behavior change [8,12]. Unlike other behavioral change interventions, mHealth interventions allow for the assessment of user engagement or intervention fidelity. If user engagement is determined to be associated with CVD health behavior change and risk factor outcomes, clinicians and providers could use summary reports of user engagement with mHealth interventions as a proxy for determining how individuals adhere to health behavior recommendations.

The process of user engagement with mHealth interventions can be measured either subjectively via self-report (eg, focus groups, observation, think-aloud activities, ecological monetary assessments, interviews, and questionnaires) to capture the emotional and cognitive experiences or objectively via physiological measurements or app analytics (eg, ecological monetary assessments, eye tracking, time spent on a page, revisits to app) to capture the behavioral experiences [9-11]. When measuring user engagement with smartphone apps, the more relevant measures include focus groups, interviews, questionnaires, ecological monetary assessments, and app analytics. App analytics measure the behavioral manifestations of user engagement with smartphone apps and can be divided into intersession and intrasession measures [11]. Intersession measures assess long-term user engagement with smartphone apps across multiple sessions [11]. Intrasession measures assess user engagement with smartphone apps within a single session [11]. For this systematic review, the emotional and cognitive aspects of user engagement are operationalized through questionnaires and the behavioral aspects of user engagement through app analytics.

There has been considerable research across multiple disciplines, examining what factors are associated with higher user engagement, including intervention content (feedback, goal setting, reminders, self-monitoring, and social support features), modes of content delivery (control, credibility, novelty, personalization, and professional support features), demographic characteristics (age, computer literacy, education, ethnicity, employment, and gender), and psychological characteristics (experience of well-being, mental health, motivation, and self-efficacy) [10]. However, a key question to address is whether the degree of user engagement with an mHealth intervention correlates with achieving the targeted outcomes, in this case, CVD risk factor modification and outcomes. Determining whether user engagement with smartphone apps is associated with improved CVD health behavior change and risk factor modification will be critical for determining their clinical utility in the future.

### Objectives

As smartphone ownership becomes more prevalent and individuals increasingly use their devices for health information and management, with 62% of smartphone owners found in a prior study to have used their smartphone in the past year to look up health information [6], we require a better understanding of user engagement with smartphone apps. To our knowledge, no systematic reviews have been previously conducted with the primary aim of assessing the relationship between user engagement with smartphone apps and CVD health behavior change and risk factor outcomes. Schoeppe et al [13] conducted a systematic review to evaluate the efficacy of interventions that used smartphone apps to improve diet, PA, and sedentary behavior. However, they only found 3 studies that examined the relationship between user engagement and improvements in PA and healthy eating and cited the need for additional research to examine the relationship between user engagement and the outcomes of interest [13]. Therefore, the purpose of this systematic review, conducted 4 years following the work of Schoeppe et al [13], is to determine if user engagement with smartphone apps for the prevention and management of CVD is associated with improved CVD health behavior change and risk factor outcomes.

## Methods

### Search Strategy and Eligibility Criteria

ES searched PubMed, EBSCOhost, and CINAHL for articles published in English between 2007 and 2020. The review was limited to this period, as smartphones were not available until 2007. No search limitations were placed on participant age, setting, or population. Specific limitations were not placed on the population to identify individuals enrolled along the spectrum of CVD prevention (primordial, primary, and secondary). Although no search restrictions were placed on study duration, there were restrictions placed on study design that aimed to return studies conducted using a correlational design or nested within a randomized controlled trial (RCT), quasi-experimental design, or mixed methods design.

The following keywords were used to identify candidate studies: (Disease Management OR Disease Prevention OR Obesity OR Overweight OR Weight Loss OR Heart Diseases OR Vascular Diseases OR Cardiovascular Diseases OR Coronary Artery Diseases OR Heart Failure OR Hypertension OR Diabetes OR Exercise OR Physical Activity) AND (Mobile Applications OR mHealth OR Mobile Health OR iPhone OR Android OR Smartphone) AND (Engag* OR Experienc* OR Usage OR Usability or Involv*) AND (Randomized Controlled Trial OR Non-Randomized Controlled Trial OR Evaluation Studies OR Quasi-Experimental OR Mixed-methods OR Correlation Studies). The final searches were conducted on January 28, 2020. The complete search strategy can be found in Multimedia Appendix 1. The search terms around user engagement are meant to encompass both objective and subjective experiences. Unlike the other CVD risk factors discussed previously, we decided not to include smoking in the search strategy, as its prevalence is low and decreasing among US adults. Covidence, a software for managing and streamlining the systematic review process, was used to screen the returned studies and remove duplicates.

Studies were eligible for inclusion if they (1) evaluated user engagement with a smartphone app; (2) included a smartphone app that was used by an individual to manage his or her cardiovascular health; (3) assessed CVD health behavior change or risk factor outcomes (ie, medication adherence; symptom management; and changes in diet, PA, weight, and biomarkers) for primordial, primary, or secondary prevention of CVD (hypertension, coronary artery disease, obesity, diabetes, myocardial infarction, or heart failure), not including stroke; and (4) assessed whether user engagement with a smartphone app was associated with the CVD health behavior change or risk factor outcome.

Studies were excluded if the sample size for the mHealth intervention group was less than 50 participants to reduce the likelihood of drawing conclusions from insufficiently powered studies. Although there were no specific search limitations placed on the study population, with the intention of identifying individuals enrolled along the spectrum of CVD prevention (primordial, primary, and secondary), if the study was not focused on CVD management or prevention, they were excluded. Examples of study populations excluded for this reason included those where there may have been elements of the intervention that aimed to improve cardiovascular health, but overall the focus was on improving psychological distress, chronic kidney disease management, type 1 diabetes management, stroke recovery, or fertility among couples attempting conception. The reasons for exclusion were assigned based on a specific hierarchical structure, moving from broader to more specific exclusion criteria. Coauthors progressed sequentially through the following reasons for exclusion: (1) intervention or population not related to CVD behavior change, (2) smartphone app was not used by the patient, (3) less than 50 participants in the mHealth intervention groups or with engagement data, (4) no measure of user engagement, (5) outcome not related to CVD prevention or management, and (6) relationship between user engagement and CVD outcome not assessed. Once an article met any of these exclusion criteria, the coauthors assigned that as the reason for exclusion and did not continue to assess the article for the subsequent exclusion criteria.

### Screening Process and Data Extraction

Each retrieved title and abstract were screened by ES to determine eligibility to qualify for full-text review. Occasional full-text reviews were completed when the operationalization of user engagement with the smartphone app was unclear from the abstract. The articles identified for full-text review were independently examined for inclusion by ES and JA. A consensus was reached on all the articles eligible for inclusion, and a third reviewer was not needed. ES extracted data on study and sample characteristics, intervention description, measures of user engagement with the smartphone app, and results regarding the relationship between user engagement with the smartphone app and the CVD health behavior change or risk factor outcome. In some instances, ES reviewed associated protocol papers to obtain the necessary data on study design, quality, and intervention description for the included articles. Data were extracted into a table to summarize the findings for the narrative results of this review.

In this review, if multiple treatment arms in a study used a smartphone app, the results with regard to the relationship between user engagement and the outcome of interest are presented as they were in the original study (ie, either combined or separated user engagement metrics across groups). If the relationship between user engagement and the health outcome was reported at multiple time points, the end of the treatment time point was used by default when determining if app user engagement was or was not significantly associated with the outcome of interest. In the Results section, we characterize a statistically significant association found in the desired direction between user engagement and the outcome of interest (ie, a positive association between user engagement and PA or a negative association between user engagement and weight) as a positive finding. We also label a nonstatistically significant association in the expected direction, no association, or an association in the opposite direction than expected as a negative finding. Findings that ended up not being reported were also labeled as a negative finding. ES and RP independently assessed the methodological rigor of the included studies via the Joanna Briggs Checklist for analytical cross-sectional studies [14]. A consensus was reached between these 2 authors on the methodological rigor for each study, and a third reviewer was not required. The checklist for analytical cross-sectional studies was chosen, as the focus for this review was on the relationship between user engagement and the CVD health behavior change or risk factor outcome of interest, not the difference in the outcome between intervention groups or the change in the outcome over time (ie, the correlational designs nested within the RCT or quasi-experimental designs).

## Results

### Results of the Search

**Figure 1:**
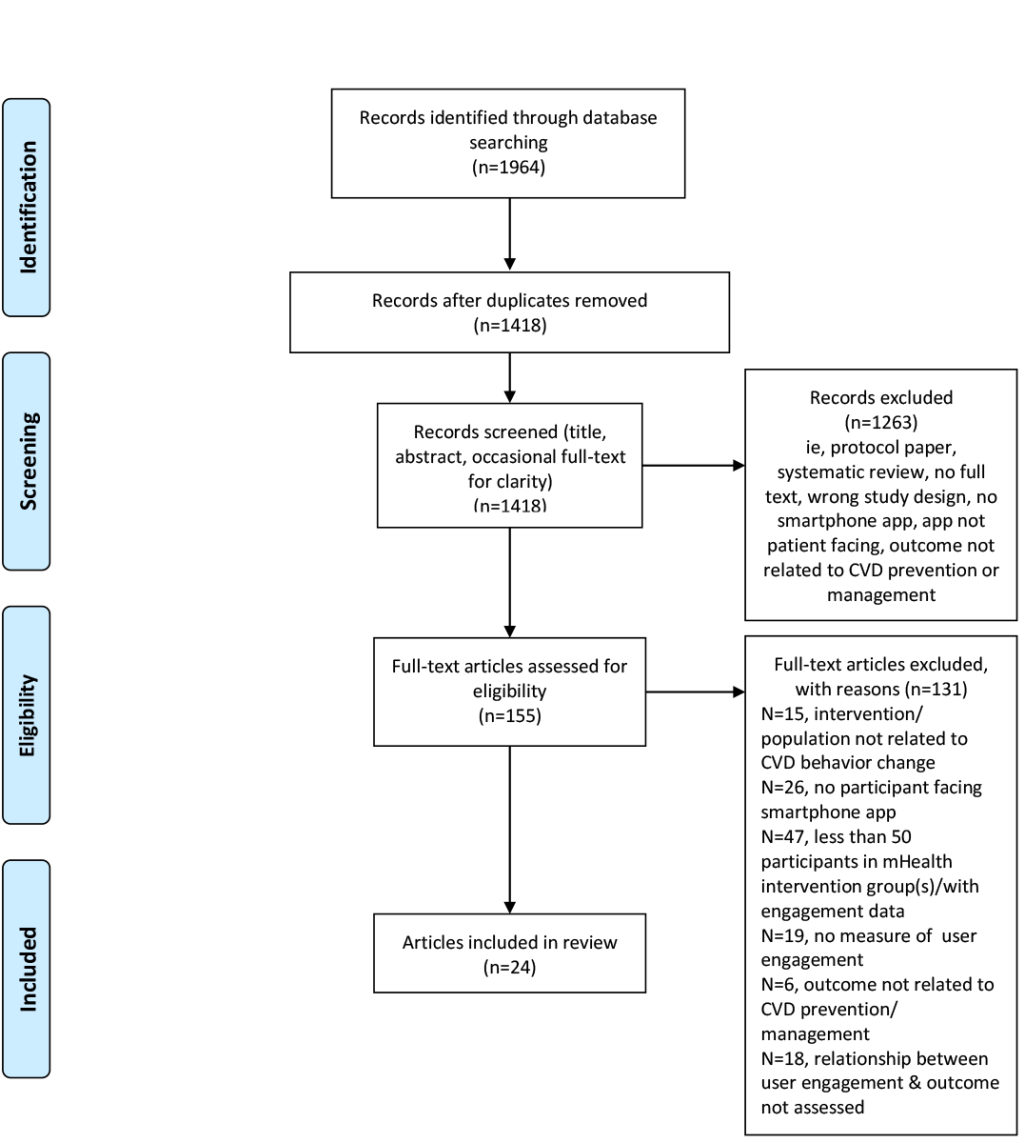
Preferred Reporting Items for Systematic Reviews and Meta-Analyses diagram depicting the flow of records. CVD: cardiovascular disease.

The PRISMA (Preferred Reporting Items for Systematic Reviews and Meta-Analyses) guidelines provided the structure for the flow of articles throughout the critical review process, which is shown in Figure 1 [15]. A total of 1964 records were identified from the 3 electronic databases. Then, 546 duplicate records were removed, and the titles and abstracts of the remaining 1418 records were reviewed. Of the 1418 records, 155 were identified for full-text review. Of 155 articles, 131 were excluded, and 24 articles (ie, studies), assessing 22 individual interventions, were deemed eligible for inclusion in this systematic review. Of the 24 studies included in this review, 16 (67%) were published in mHealth or technology journals, and 8 (33%) were published in medical or clinical journals. These findings likely reflect journal preferences, with medical and clinical journals, perhaps favoring studies that focus more on clinical outcomes and not necessarily user engagement with mHealth interventions.

### Study Characteristics

The study characteristics are summarized in Multimedia Appendix 2 [16-39]. Of the 24 studies, 17 used an RCT design [16-32], 4 used a retrospective analysis [33-36], and 3 used a single-group pre-and posttest quasi-experimental design [37-39]. Although 20 studies used an RCT or single-group pre- and-posttest quasi-experimental design, it was the correlational studies nested within these larger parent studies that were of interest for this review. For the 17 RCTs, intervention duration ranged from 6 weeks [22] to 24 months [17,24]. For the 3 single-group pre- and-posttest quasi-experimental studies, intervention duration ranged from 3 months [37] to 6 months [38,39]. For the studies using a prospective design, follow-up ranged from 1 month [25,26,28] to 24 months [17,24]. All 24 studies were conducted in developed countries, including the United States (n=11), Australia (n=3), Canada (n=2), Spain (n=2), the United Kingdom (n=1), Norway (n=1), Finland (n=1), Japan (n=1), Singapore (n=1), and Korea (n=1). Overall, 2 studies with the same first author assessed one intervention (MyFitnessPal) [25,26], and another 2 studies with the same first author also assessed one intervention (Noom) [33,34]. Therefore, although there were 24 studies, only 22 individual interventions were assessed.

Of the 17 RCT studies, 14 conducted an a priori power analysis. Of these 14 RCTs, 3 detected a significant difference in the primary outcome between groups [21,23,27], 9 did not detect a significant difference in the primary outcome between groups [16,17,22,24-26,29,31,32], and 2 did not report the results of the difference in the primary outcome between groups [18,30]. One of the studies that did not detect a significant difference between groups did not achieve the target sample size [32]. Three RCTs did not conduct an a priori power analysis [19,20,28], one of which was a pilot study [28]. However, one study still detected a significant difference in the primary outcome between groups [19]. Among the RCTs, only one study conducted a post hoc power analysis for the relationship between user engagement and the outcomes of interest [20]. None of the 3 single-group pre- and-posttest quasi-experimental studies conducted a power analysis [37-39]. One study had a very large sample size and found a significant difference in the primary outcome over time [37]. The other 2 were pilot studies and did not conduct inferential statistics regarding the primary outcome [38,39]. None of the 4 retrospective studies conducted a power analysis [33-36]. One study detected a significant difference in the primary outcome over time [36]; however, the other 3 studies did not report differences in the primary outcome over time [33-35].

Overall sample sizes ranged from 55 [38] to 324,649 participants [35]. Although this review was not limited to a specific age group, all eligible studies comprised adults, but one study enrolled participants as young as 16 years [28]. All 24 studies reported on sex, with 16 of the samples consisting largely of women (range 51%-91%) [22,38], 7 of the samples consisting largely of men (range 51%-100%) [16,21], and one split evenly [39]. Thirteen studies reported sample races [17-19,21-28,32,39], with 7 consisting largely of White participants [18,19,22,24-26,28]. Seventeen studies reported on sample educational level, with the majority of the participants within each individual study having attended some college [16-26,28-32,38,39]. Ten studies limited their sample to participants who were overweight or obese [17,19,21-26,28,35], and another 11 studies reported enrolling participants with a baseline mean or majority percentage BMI indicating overweight or obesity [16,20,22,27,29-31,33,34,38,39]. The majority of the studies recruited participants from a community setting, except for 5 studies that recruited participants from hospitals [21], a hospital-based diabetes management education program [32], primary care [23,29], and a community health care facility [39].

All 22 interventions assessed included an app for addressing CVD risk factors. Of 22 interventions, 11 (50%) included commercial apps [19,20,22,25-27,32-37,39] and 11 (50%) included investigator-developed apps [16-18,21,23,24,28-31,38]. In addition, 73% (16/22) of the interventions consisted of not only an app but also other components, including in-person meetings, emails, text messaging, phone consults, websites, Facebook groups, blogs, and podcasts [16-26,29-31,36,38,39]. Of 22 interventions, 6 (27%) consisted solely of an app [27,28,32-35,37]. Twelve apps were also paired with tracking devices (ie, pedometers, weight scales, glucose meters) [18,20-24,29-31,35,38,39]. Most of the studies assessed weight loss interventions, with 27% (6/22) of the interventions addressing additional CVD risk factors, including type 2 diabetes, sleep, stress, excessive alcohol consumption, and smoking [18,20,31,32,36,39]. The interventions focused on weight loss used a variety of strategies, including nutrition and PA tracking-energy expenditure, diet and exercise education, podcasts, social support, recipes, and behavior change techniques.

### User Engagement With Smartphone App Measures

The behavioral manifestations of user engagement with smartphone apps were assessed using app analytics. Only intersession measures were used in the included studies. In 3 of the 17 studies that combined other intervention strategies with the app (ie, participants had the choice to use both the app and other intervention strategies such as a website), they did not differentiate app from website user engagement and provided a combined effect [16,17,20]. The intersession measures used were general usage and overall self-monitoring over a defined period. The general usage of the interventions was assessed in 11 studies through multiple means, including tracking the number of log-ins per week or month, number of usage days, total time spent using the app, and number of times the different app features were used [16,20,24,28-32,36-38]. In 2 studies, self-reported weekly app use was assessed as a proxy for intersession general usage measures [19,22]. In 14 of the studies, researchers assessed overall self-monitoring (eg, total number of days self-monitoring or entries made in the app) [16-18,21,23-27,32-35,39]. User engagement with the smartphone apps was also categorized by frequency and pattern of self-monitoring or general usage in 14 of the studies [17,19-21,23,27,29-31,35-39].

In 33% (8/24) of the studies, there was no indication of how frequently participants were able to or expected to engage with the intervention (ie, intended engagement) [18-20,28,31,32,35,36]. In 67% (16/24) of the studies, there was some indication of how frequently participants were able to or expected to engage with the intervention [16,17,21-27,29,30,33,34,37-39], whether it was broad instructions to self-monitor behaviors daily or almost daily [16,17,22,25,26,29,30,33,34,37,38], multiple times a day [21,27], or more specific recommendations for each feature in the intervention [23,24,39]. It was not always clear in the studies that indicated participants were able to self-monitor within the intervention daily if they were explicitly told or prompted to do so.

Overall, 6 studies included a smartphone app in more than one treatment arm [22,24-26,30,31]. In 2 of these studies, where there was no difference in the smartphone app between the 2 groups, user engagement data across both groups with the smartphone app were combined when assessing the relationship between user engagement and the outcome of interest [22,31]. In another study, where the versions of the smartphone app were different between the 2 arms (gamified app vs nongamified app), the app user engagement data were also combined across groups [29]. In studies assessing the MyFitnessPal app, user engagement was both combined and separated across groups when assessing the relationship between user engagement and the outcome of interest [25,26]. As the app did not differ across groups, but rather instruction on how to engage with the app as well as supplementary material, in the narrative of this review, we present the combined results [25,26]. In another study, where both intervention arms had access to the same app, one without reminders and prompts, the results were presented separately for each arm [24]. Multimedia Appendix 2 provides a description of how user engagement with the smartphone apps was operationalized in each study. None of the studies reported on the subjective experience of user engagement [11] with a smartphone app; however, a few did report on satisfaction and usability of the app [9].

### Quality of Studies

Of the 24 included studies, 21 used convenience or purposive sampling [16-28,30-32,35-39] and only 3 used random sampling [29,33,34], limiting the external validity of these results. Seventeen of the studies used an RCT design [16-32], one of which also used random sampling [29], strengthening the internal validity. In Multimedia Appendix 3, the methodological rigor for each study is presented using the Joanna Briggs Checklist for analytical cross-sectional studies [14]. The rules for how the studies were scored on methodological rigor for each question, directed by the Joanna Briggs Checklist for analytical cross-sectional studies guidelines, are also provided in Multimedia Appendix 3.

The questions that studies performed the worst on the Joanna Briggs Checklist for analytical cross-sectional studies were question 1 (“Were the criteria for inclusion in the sample clearly defined?”) in which 42% (10/24) of the studies received a score of yes, question 3 (“Was the exposure measured in a valid and reliable way?”) in which none of the studies received a score of yes, question 5 (“Were confounding factors identified?”) in which 42% (10/24) of the studies received a score of yes, and question 8 (“Was appropriate statistical analysis used?”) in which 38% (9/24) of the studies received a score of yes. The studies performed well on question 2 (“Were the study subjects and the setting described in detail?”) in which 71% (17/24) of the studies received a score of yes, question 4 (“Were objective, standard criteria used for measurement of the condition?”) in which 94% (17/18, studies with a score of not applicable not included in denominator) of the studies received a score of yes, and question 7 (“Were the outcomes measured in a valid and reliable way?”) in which 88% (21/24) of the studies received a score of yes.

Of the studies that were not nested within an RCT, 5 found positive results [33-37], one demonstrated more mixed results [39], and one with a smaller sample size (n=55) found negative results [38]. Thus, it does not appear as though studies nested within a poorer quality design demonstrated more negative results. In general, the sample sizes were large (88% had a sample size ≥100 participants). Heterogeneous app analytics were used across studies to assess user engagement with smartphone apps, making it difficult to draw strong conclusions. In addition, in 3 studies, user engagement was operationalized across technology platforms, limiting the accurate assessment of the relationship between user engagement with the app and the outcome [16,17,20].

### Relationship Between User Engagement With Smartphone Apps and Health Outcome

Multimedia Appendix 2 also provides a description of the relationship between user engagement with smartphone apps and the CVD health behavior change or risk factor outcome or both in each study. If the relationship between user engagement and the health outcome was reported at multiple time points, the end of the treatment time point was used by default when determining whether app user engagement was or was not significantly associated with the outcome of interest. There were, however, 2 studies that presented these findings at only a preliminary [32] or longer follow-up [29] time point; thus, the findings at these time points had to be used instead when making a determination.

#### Changes in Anthropometrics

Overall, 15 studies assessed the relationship between user engagement with a smartphone app and changes in anthropometrics, including weight, BMI, percent body fat, and waist circumference.

##### User Engagement and Change in Weight

Thirteen studies assessed the relationship between user engagement with a smartphone app and change in weight. Nine studies reported statistically significant greater weight loss with higher user engagement with a smartphone app, with follow-up ranging from 2 to 12 months [19-21,23,25-27,34,35]. Both general usage [19-21] and self-monitoring measures [23,25-27,34,35] of user engagement with a smartphone app were used. In particular, entering PA, dietary behaviors, and weight; more frequent upload of meal photographs; completing more educational articles; customizing more features; and posting on social platforms were significantly associated with greater weight loss [23,25-27,34,35]. However, simply commenting on other users’ posts was not [34]. Four studies that assessed the relationship between user engagement with a smartphone app and weight demonstrated negative results, with follow-up ranging from 3 to 24 months [17,24,28,39]. Self-monitoring measures [17,24,39] and general usage measures [24,28] of user engagement with smartphone apps were used. Although Lin et al [24] did not find statistically significant correlations between user engagement and weight change at 24 months (the length of intervention duration), at 6 months, both intervention arms with an app found that as app user engagement increased, weight decreased. In addition, at 12 months, this relationship remained statistically significant for the app intervention arm paired with group dietitian–led sessions and phone calls [24].

##### User Engagement and Change in BMI

Four studies assessed the relationship between user engagement with a smartphone app and BMI [18,20,21,33]. Both general usage [20,21] and self-monitoring measures [18,33] of user engagement with smartphone apps were used. Three of these studies reported a statistically significant greater reduction in BMI with higher user engagement with the smartphone app, with follow-up ranging from 6 to 12 months [20,21,33]. In particular, activities within the Noom weight loss app, including logging food and group participation (number of original posts and comments and likes on others’ posts), were significantly associated with a reduction in BMI [33]. Among Gray Matters app users, there was no correlation between the average number of log-ins per day and change in BMI at 6 months [18].

##### User Engagement and Change in Waist Circumference and Body Fat Percentage

Three [20,21,27] and 2 [20,21] studies assessed the relationship between user engagement and waist circumference and body fat, respectively. In 2 of the studies, sustained users of the app and web technology interventions and more frequent meal photograph uploads were significantly associated with greater reduction in percent body fat [20] and waist circumference [20,27], with follow-up ranging from 2 to 12 months. One study that aimed to assess the relationship between user engagement and waist circumference and percent body fat at 6 months did not report the data [21].

#### CVD Health Behavior Change

Six studies assessed the relationship between user engagement with a smartphone app and change in PA or dietary behaviors or both [16,22,29,30,37,38], and one study assessed the relationship between app user engagement and diabetes self-care behaviors [32]. The changes in health behavior were collected via self-reported data entered into the app or through paired devices [16,22,29,30,37,38] or via surveys [16,29,30,32]. General usage [16,29,30,37,38] and self-monitoring measures [16,22] of user engagement with smartphone apps were used.

##### User Engagement and Change in PA

Among the 5 studies that assessed change in objectively measured PA (ie, step count and minutes of moderate to vigorous physical activity) [22,29,30,37,38], 3 studies found statistically significant associations between user engagement and increases in objectively measured PA with follow-up ranging from 3 months to 12 months [29,30,37]. Two of the 5 studies with smaller sample sizes (n=67 and n=55) and follow-up at 6 weeks and 6 months did not [22,38]. The relationship between user engagement with smartphone apps and self-reported PA was assessed across 3 studies with follow-up ranging from 3 to 12 months [16,29,30]. One study found that increased user engagement was associated with increased self-reported PA [30], and 2 studies did not [16,29]. In a study conducted by Edney et al [30], app engagement data were combined across multiple treatment arms using a gamified versus nongamified app. Although the results for the relationship between user engagement and step count and self-reported PA were not presented separately for each arm, they did report that gamified app users were more likely to be in the high user engagement group [30].

##### User Engagement and Change in Dietary and Diabetes Self-Care Behaviors

In 2 studies with follow-up ranging from 9 to 12 months, no general usage or self-monitoring measures of user engagement with the intervention were associated with a change in self-reported dietary behaviors [16,29]. One study that conducted an exploratory analysis assessing the relationship between overall app use and self-reported diabetes self-care behaviors at 3 months did not find a statistically significant association [32].

#### Change in Risk Factors and Biomarkers

Eight studies assessed the relationship between user engagement with a smartphone app and biomarkers, including hemoglobin A_1c_ (HbA_1c_; n=5), heart rate (n=1), systolic blood pressure (SBP) and diastolic blood pressure (DBP; n=2), cholesterol (n=2), triglycerides (n=2), blood carotenoids (n=1), serum glucose (n=2), and insulin levels (n=1) [18,20,21,27,31,32,36,39]. General usage [18,20,31,32] and self-monitoring measures [18,21,27,32] of user engagement with smartphone apps were used.

##### User Engagement and Change in HbA_1c_

Five studies assessed the relationship between user engagement with a smartphone app and HbA_1c_ [27,31,32,36,39]. Three of these studies found that general usage of the app overall was significantly associated with a decrease in HbA_1c_ with follow-up ranging from 3 to 12 months [31,32,36]. However, when assessing the relationship between self-monitoring measures and decrease in HbA_1c_, the findings were slightly more mixed. One study found that more frequent meal photograph uploads, when comparing the highest tertile to the lowest tertile, were significantly associated with decreased HbA_1c_ at 8 weeks [27], but 3 other studies found that meal or diet tracking was not associated with a decrease in HbA_1c_ with follow-up ranging from 3 to 12 months [31,32,39]. In addition, greater use of the exercise features in one study was associated with a decrease in HbA_1c_ at 3 months [32] but not in another study at 12 months [31]. Weight tracking [39], but not blood glucose tracking [31,32,39], was found to have a significant relationship with a decrease in HbA_1c_.

##### User Engagement and Change in Blood Pressure, Lipid Panel, and Other Biomarkers

Among the 3 other studies assessing the relationship between user engagement with a smartphone app and risk factors and biomarkers, one found mixed results [18] and the other 2 found negative results [20,21]. Among participants enrolled with the Gray Matters app (n=104), there was a statistically significant positive correlation between the average number of health behavior questions answered per day and improvement in total and high-density lipoprotein (HDL) cholesterol at 6 months [18]. However, the average number of logs completed per day was not significantly associated with resting heart rate, SBP, DBP, cholesterol, triglycerides, blood carotenoids, serum glucose, or insulin levels at 6 months [18]. Among users of an app and web-based technology intervention (n=118), there were no statistically significant relationships between sustained and nonsustained usage and a change in aerobic fitness (METmax), SBP, DBP, triglycerides, or total cholesterol at 12 months [20]. Among users of the SmartCare weight loss app, at 6 months, there were no statistically significant differences in lipid panel improvement (total cholesterol, HDL cholesterol, and triglycerides) between those who entered anthropometric data at least 3 times per week versus those who did so less than 3 times per week [21].

Table 1 provides the number of studies that looked at each outcome, the number of studies for each outcome that had a positive finding, and the number of studies for each outcome that had a negative finding. A positive finding refers to a statistically significant association found in the desired direction between user engagement and the outcome of interest (ie, a positive association between user engagement and step count or a negative association between user engagement and weight). A negative finding refers to a nonstatistically significant association in the expected direction, no association, or an association in the opposite direction than expected. None of the studies in this review found a significant association in the direction opposite to what was expected. For the study that ended up not reporting waist circumference and body fat percentage results [21], this was also categorized as a negative finding.

**Table 1:** Findings for the relationship between user engagement and the cardiovascular disease health behavior change or health outcome.

Outcome	Studies with positive finding, n (%)	Studies with negative finding, n (%)
Weight (n=13)	9 (69)	4 (31)
BMI (n=4)	3 (75)	1 (25)
Percent body fat (n=2)	1 (50)	1 (50)
Waist circumference (n=3)	2 (67)	1 (33)
Objectively measured physical activity (n=5)	3 (60)	2 (40)
Self-reported physical activity (n=3)	1 (33)	2 (67)
Self-reported diet (n=2)	0 (0)	2 (100)
Hemoglobin A_1c_^a^ (n=5)	3 (60)	2 (40)
Systolic blood pressure (n=2)	0 (0)	2 (100)
Diastolic blood pressure (n=2)	0 (0)	2 (100)
Total cholesterol (n=3)	0 (0)	3 (100)
HDL^b^ cholesterol (n=1)	0 (0)	1 (100)
Triglycerides (n=3)	0 (0)	3 (100)
Resting heart rate (n=1)	0 (0)	1 (100)
Blood carotenoids (n=1)	0 (0)	1 (100)
Serum glucose (n=1)	0 (0)	1 (100)
Insulin levels (n=1)	0 (0)	1 (100)
METmax (n=1)	0 (0)	1 (100)

^a^HbA_1c_: hemoglobin A_1c_.

^b^HDL: high-density lipoprotein.

A meta-analysis was not conducted because the primary objective of this systematic review was not to assess the effectiveness of the interventions as a whole but rather, to determine whether increased app user engagement was associated with improvement in CVD health behavior change and risk factor outcomes. In addition, the heterogeneity in study designs and methods would have led to bias in a meta-analysis. However, as a sensitivity analysis, we assessed the relationship between user engagement with a stand-alone app versus an app plus other intervention components and weight, the most frequently assessed CVD health outcome. Of the 7 studies that assessed a stand-alone app [27,28,32-35,37], 4 assessed the relationship between user engagement and weight [27,28,34,35]. Of these 4 studies, 3 (75%) found positive results [27,34,35]. Of the 17 studies that assessed an app plus other intervention components, 9 assessed the relationship between user engagement and weight [17,19-21,23-26,39]. Of these 9 studies, 6 (67%) found positive results. Thus, in this sensitivity analysis, a higher percentage of studies assessing the relationship between user engagement with a stand-alone app and weight found positive results. However, the number of studies assessing a stand-alone app that also examined the relationship between user engagement and weight was small.

## Discussion

### Principal Findings

We found 24 studies that met the eligibility criteria for this systematic review. This systematic review revealed that increased user engagement with a smartphone app, measured by general usage or self-monitoring entries or both, for the prevention and management of CVD may be associated with a reduction in weight (9/13 studies with positive findings) and BMI (3/4 studies with positive findings). Although only a few studies assessed the relationship between user engagement with a smartphone app and body fat percentage (1/2 studies with positive findings), waist circumference (2/3 studies with positive findings), and objectively measured PA (3/5 studies with positive findings), the findings were generally positive. However, although only a few studies assessed the relationship between user engagement with a smartphone app and dietary (2 studies) and diabetes self-care behaviors (1 study), the results were all negative. Among the 5 studies that assessed the relationship between user engagement with a smartphone app and HbA_1c_, the results were promising (3/5 studies with positive findings). The 3 studies that assessed the relationship between user engagement with a smartphone app and the remaining biomarkers were largely statistically nonsignificant.

There are multiple explanations for why higher user engagement was associated with greater weight loss but not with reduction in biomarkers, such as blood pressure, total cholesterol, and triglycerides. First, the 3 studies that looked at these outcomes were likely underpowered to detect a significant relationship. In fact, Mattila et al [20] conducted post hoc analyses demonstrating that they were underpowered to detect significant associations between user engagement and change in SBP, DBP, total cholesterol, and triglycerides, although they were adequately powered to detect significant associations between user engagement and change in weight, BMI, waist circumference, and body fat percentage. The study by Hartin et al [18] was only powered to detect a medium effect size of 0.50 difference between the 2 treatment groups on SBP, DBP, total cholesterol, and triglycerides. The study by Oh et al [21] was powered to detect a 1.81 kg difference in weight between the 2 treatment groups, but they did not conduct power analyses for the other biomarker outcomes.

Second, only one of these 3 studies [21] incorporated a medication adherence component into the intervention. This intervention incorporated telephone consultations with nurses, exercise specialists, and clinical dietitians, which could include discussion regarding medications, as well as covering the costs of medications as an incentive to partake in the study [21]. However, it is unclear how frequently medication adherence was discussed as a part of these consultations. The other 2 studies did not include medication adherence components in their intervention [18,20]. It is possible that the lifestyle interventions in these 3 studies, largely focused on diet and PA, were not sufficient to significantly reduce these CVD biomarkers. In the future, researchers should consider adding education on CVD medications such as antihypertensives and statins as well as medication adherence self-monitoring capabilities into their mHealth interventions if they want to impact CVD biomarkers.

### Comparison With Prior Work

Few systematic reviews have been conducted in this area, with most assessing the effectiveness of interventions on health outcomes or strategies to promote user engagement with mHealth interventions, as opposed to directly assessing the relationship between user engagement with mHealth and CVD health behavior change and risk factor outcomes. Semper et al [40] included 6 studies in their systematic review assessing the effectiveness of smartphone apps, which encourage dietary self-regulatory strategies, for weight loss among overweight and obese adults. This review demonstrated that participants using the smartphone apps in all studies lost at least some weight; however, when compared with other self-monitoring tools, there was no significant difference in the amount of weight loss [40]. Semper et al [40] did not report on user engagement with the apps. Although studies such as this have demonstrated that self-monitoring may be associated with greater weight loss, this does not provide a comprehensive picture of how user engagement with smartphone apps is associated with health behavior change and outcomes. Smartphone apps are capable of incorporating multiple behavior change strategies, such as goal setting, feedback, reminders, and social support features, and simply assessing self-monitoring of health behaviors may not be indicative of user engagement with the app as a whole. Thus, in this review, we build upon prior research by assessing user engagement with the app as a whole as well as individual features such as self-monitoring.

Schoeppe et al [13] conducted a systematic review to evaluate the efficacy of interventions that used smartphone apps to improve diet, PA, and sedentary behavior. Of the 23 included studies among adults, 17 demonstrated significant improvements in PA (n=13), diet (n=6), weight (n=4), blood pressure (n=2), sedentary behavior (n=1), fitness (n=1), and cholesterol (n=1) [13]. Eleven of the studies included in this review reported app usage to assess user engagement [13]. However, only 3 of these studies examined the association between app usage and changes in behavior and health outcomes, cautiously demonstrating that higher app usage was associated with improvements in PA and healthy eating [13]. The authors of this review recommended that further work be conducted to examine the relationship between user engagement and the outcomes of interest [13]. This systematic review fills this gap in the literature by building upon these findings and examining the relationship between user engagement and additional behavior change and health outcomes.

Another review assessed factors related to user engagement with internet behavioral interventions across many chronic health conditions, including type 2 diabetes, weight loss maintenance, and CVD [41]. They found that the interventions that adapted to individual needs, including record keeping, personalized feedback, and accountability, were more engaging [41]. In this systematic review, measures of user engagement with a smartphone app, such as self-monitoring (record keeping), were also frequently associated with improved risk factor outcomes, including a reduction in weight and BMI. Another systematic review of 14 RCTs looked at the effectiveness of technology-based strategies (eg, offering digital health intervention assistance) to promote engagement with digital health interventions (web-based platforms paired with emails, telephone calls, and texting) that targeted various health behaviors and conditions [42]. The studies often reported small-to-moderate effects of technology-based strategies on engagement compared with no strategy [42]. Previous reviews have focused on compiling strategies to promote user engagement with mHealth interventions. This systematic review builds upon previous work by assessing the relationship between user engagement with smartphone apps and the actual CVD health behavior change or risk factor outcome.

### Limitations and Future Directions

The studies included in this review varied in how they operationalized and analyzed user engagement with smartphone apps, making it challenging to compare results across studies. None of the studies used intrasession measures of user engagement with a smartphone app, limiting this review to intersession measures. Intersession measures provide a better understanding of long-term user engagement with smartphone apps, but intrasession measures could provide valuable insight into how users engage within a single session. Researchers should consider using both types of measures. In addition, researchers should obtain both general usage and self-monitoring intersession measures. General usage measures will make it easier to compare results across studies, but self-monitoring activities within the app can provide more insight into specific intervention component use and whether engagement with them is associated with better outcomes.

In addition, 33% (8/24) of the studies in this review did not provide any indication of intended engagement with the intervention. Among the 67% (16/24) of the studies that did indicate how frequently participants were able to use certain features, it was not always clear whether participants were explicitly told or prompted on how frequently to engage. In the future, providing clear instructions for intended engagement and then determining whether not meeting, meeting, or exceeding intended engagement expectations are associated with achieving the intended outcomes will facilitate advancement in this field. Ultimately, seeking to determine what is considered sufficient user engagement with the intervention to achieve the intended outcomes (ie, *effective engagement*), as opposed to the current standard, that more user engagement is always better [8,10]. No studies have assessed the subjective experience of user engagement with the smartphone app, largely limiting this review to the behavioral manifestations of user engagement with the smartphone app via app analytics. This narrow focus on the behavioral aspects of user engagement is reflective of the state of the science but is not sufficient to assess the multidimensional concept of user engagement. Future studies could consider using the User Engagement Scale [43] or the eHealth Engagement Scale [44] to assess the subjective experience of user engagement.

The majority of the assessed apps focused on weight management. Additional studies are needed to assess user engagement with smartphone apps targeting hypercholesterolemia, diabetes, and hypertension, other important risk factors for CVD. In addition, most studies recruited participants from the community setting. In the future, more studies need to be conducted where participants are recruited in the inpatient or outpatient setting, and the apps are integrated with clinical care to determine whether this further affects the relationship between user engagement with smartphone apps and CVD health behavior change and risk factor outcomes.

There are also limitations specific to the conduction of this review, which should be taken into consideration. First, hand searching was not conducted as part of the search strategy. Second, only one author reviewed the titles and abstracts. However, if there was any question of whether a study should be included or excluded at this stage, it was progressed to full-text review, at which point 2 authors assessed the potentially eligible studies. Third, only one author extracted the data from the included studies to populate the table and results. Finally, it was outside the scope of the review to report on actual user engagement outcomes; however, this may be important for future reviews.

This systematic review also has many strengths. First, we searched multiple databases starting from when smartphones first became available. Second, 2 authors independently screened the full-text articles. Third, 2 authors independently assessed the quality of the included studies using a standardized assessment tool. Fourth, we reported the study findings on the relationship between both general usage and self-monitoring measures and the outcome of interest. Fifth, we provided clear delineation of the number of studies that had a positive or negative finding for each outcome via a table.

### Conclusions

This systematic review found that user engagement with smartphone apps may be associated with risk factor outcomes, including reduction in weight and BMI, among adults using a smartphone app for CVD prevention and management. To draw stronger conclusions moving forward and to move toward the concept of *effective engagement*, the mHealth community needs to reach a consensus on how best to consistently operationalize user engagement with smartphone apps across multiple platforms and incorporate intended engagement with the intervention into measurement approaches.

## Appendix

Multimedia Appendix 1 Search strategy.

Multimedia Appendix 2 Study characteristics.

Multimedia Appendix 3 Quality assessment of studies included in this systematic review using the Joanna Briggs Checklist for analytical cross-sectional studies.

## Abbreviations

AHA: American Heart Association
CVD: cardiovascular diseases
DBP: diastolic blood pressure
HbA_1c_: hemoglobin A_1c_
HDL: high-density lipoprotein
mHealth: mobile health
PA: physical activity
RCT: randomized controlled trial
SBP: systolic blood pressure

## References

[1] Benjamin EJ, Virani SS, Callaway CW, Chamberlain AM, Chang AR, Cheng S, Chiuve SE, Cushman M, Delling FN, Deo R, de Ferranti SD, Ferguson JF, Fornage M, Gillespie C, Isasi CR, Jiménez MC, Jordan LC, Judd SE, Lackland D, Lichtman JH, Lisabeth L, Liu S, Longenecker CT, Lutsey PL, Mackey JS, Matchar DB, Matsushita K, Mussolino ME, Nasir K, O'Flaherty M, Palaniappan LP, Pandey A, Pandey DK, Reeves MJ, Ritchey MD, Rodriguez CJ, Roth GA, Rosamond WD, Sampson UK, Satou GM, Shah SH, Spartano NL, Tirschwell DL, Tsao CW, Voeks JH, Willey JZ, Wilkins JT, Wu JH, Alger HM, Wong SS, Muntner P, American Heart Association Council on Epidemiology Prevention Statistics Committee Stroke Statistics Subcommittee (2018). Heart disease and stroke statistics-2018 update: a report from the American heart association. Circulation.

[2] Burke LE, Ma J, Azar KM, Bennett GG, Peterson ED, Zheng Y, Riley W, Stephens J, Shah SH, Suffoletto B, Turan TN, Spring B, Steinberger J, Quinn CC (2015). Current science on consumer use of mobile health for cardiovascular disease prevention: a scientific statement from the American heart association. Circulation.

[3] (2018). Mobile Fact Sheet.

[4] Krebs P, Duncan DT (2015). Health app use among US mobile phone owners: a national survey. JMIR Mhealth Uhealth.

[5] (2012). Mobile Health 2012. Pew Research Center.

[6] (2015). U.S. Smartphone Use in 2015. Pew Research Center.

[7] Shan R, Ding J, Plante TB, Martin SS (2019). Mobile health access and use among individuals with or at risk for cardiovascular disease: 2018 health information national trends survey (hints). J Am Heart Assoc.

[8] Yardley L, Spring BJ, Riper H, Morrison LG, Crane DH, Curtis K, Merchant GC, Naughton F, Blandford A (2016). Understanding and promoting effective engagement with digital behavior change interventions. Am J Prev Med.

[9] Short CE, DeSmet A, Woods C, Williams SL, Maher C, Middelweerd A, Müller AM, Wark PA, Vandelanotte C, Poppe L, Hingle MD, Crutzen R (2018). Measuring engagement in eHealth and mHealth behavior change interventions: viewpoint of methodologies. J Med Internet Res.

[10] Perski O, Blandford A, West R, Michie S (2017). Conceptualising engagement with digital behaviour change interventions: a systematic review using principles from critical interpretive synthesis. Transl Behav Med.

[11] Lalmas M, O'Brien H, Yom-Tov E (2014). Measuring user engagement. Synth Lect Inform Concepts Retr Serv.

[12] O'Brien HL, Toms EG (2008). What is user engagement? A conceptual framework for defining user engagement with technology. J Am Soc Inf Sci.

[13] Schoeppe S, Alley S, Van Lippevelde  W, Bray NA, Williams SL, Duncan MJ, Vandelanotte C (2016). Efficacy of interventions that use apps to improve diet, physical activity and sedentary behaviour: a systematic review. Int J Behav Nutr Phys Act.

[14] (2017). The Joanna Briggs Institute Critical Appraisal Tools for Use in JBI Systematic Reviews Checklist for Analytical Cross Sectional Studies. Joanna Briggs Institute.

[15] Moher D, Liberati A, Tetzlaff J, Altman DG, PRISMA Group (2009). Preferred reporting items for systematic reviews and meta-analyses: the PRISMA statement. Ann Intern Med.

[16] Duncan M, Vandelanotte C, Kolt GS, Rosenkranz RR, Caperchione CM, George ES, Ding H, Hooker C, Karunanithi M, Maeder AJ, Noakes M, Tague R, Taylor P, Viljoen P, Mummery WK (2014). Effectiveness of a web- and mobile phone-based intervention to promote physical activity and healthy eating in middle-aged males: randomized controlled trial of the ManUp study. J Med Internet Res.

[17] Godino JG, Merchant G, Norman GJ, Donohue MC, Marshall SJ, Fowler JH, Calfas KJ, Huang JS, Rock CL, Griswold WG, Gupta A, Raab F, Fogg BJ, Robinson TN, Patrick K (2016). Using social and mobile tools for weight loss in overweight and obese young adults (Project SMART): a 2 year, parallel-group, randomised, controlled trial. Lancet Diabetes Endocrinol.

[18] Hartin PJ, Nugent CD, McClean SI, Cleland I, Tschanz JT, Clark CJ, Norton MC (2016). The empowering role of mobile apps in behavior change interventions: the gray matters randomized controlled trial. JMIR Mhealth Uhealth.

[19] Johnston CA, Rost S, Miller-Kovach K, Moreno JP, Foreyt JP (2013). A randomized controlled trial of a community-based behavioral counseling program. Am J Med.

[20] Mattila E, Orsama A, Ahtinen A, Hopsu L, Leino T, Korhonen I (2013). Personal health technologies in employee health promotion: usage activity, usefulness, and health-related outcomes in a 1-year randomized controlled trial. JMIR Mhealth Uhealth.

[21] Oh B, Yi G, Han MK, Kim JS, Lee CH, Cho B, Kang HC (2018). Importance of active participation in obesity management through mobile health care programs: substudy of a randomized controlled trial. JMIR Mhealth Uhealth.

[22] Wang JB, Cataldo JK, Ayala GX, Natarajan L, Cadmus-Bertram LA, White MM, Madanat H, Nichols JF, Pierce JP (2016). Mobile and wearable device features that matter in promoting physical activity. J Mob Technol Med.

[23] Bennett GG, Steinberg D, Askew S, Levine E, Foley P, Batch BC, Svetkey LP, Bosworth HB, Puleo EM, Brewer A, DeVries A, Miranda H (2018). Effectiveness of an app and provider counseling for obesity treatment in primary care. Am J Prev Med.

[24] Lin P, Grambow S, Intille S, Gallis JA, Lazenka T, Bosworth H, Voils CL, Bennett GG, Batch B, Allen J, Corsino L, Tyson C, Svetkey L (2018). The association between engagement and weight loss through personal coaching and cell phone interventions in young adults: randomized controlled trial. JMIR Mhealth Uhealth.

[25] Patel ML, Hopkins CM, Brooks TL, Bennett GG (2019). Comparing self-monitoring strategies for weight loss in a smartphone app: randomized controlled trial. JMIR Mhealth Uhealth.

[26] Patel ML, Hopkins CM, Bennett GG (2019). Early weight loss in a standalone mHealth intervention predicting treatment success. Obes Sci Pract.

[27] Tanaka K, Sasai H, Wakaba K, Murakami S, Ueda M, Yamagata F, Sawada M, Takekoshi K (2018). Professional dietary coaching within a group chat using a smartphone application for weight loss: a randomized controlled trial. J Multidiscip Healthc.

[28] van Beurden SB, Smith JR, Lawrence NS, Abraham C, Greaves CJ (2019). Feasibility randomized controlled trial of ImpulsePal: smartphone app-based weight management intervention to reduce impulsive eating in overweight adults. JMIR Form Res.

[29] Garcia-Ortiz L, Recio-Rodriguez JI, Agudo-Conde C, Patino-Alonso MC, Maderuelo-Fernandez J, Repiso Gento I, Puigdomenech Puig E, Gonzalez-Viejo N, Arietaleanizbeaskoa MS, Schmolling-Guinovart Y, Gomez-Marcos MA, Rodriguez-Sanchez E, EVIDENT Investigators Group, Mobilizing Minds Research Group (2018). Long-term effectiveness of a smartphone app for improving healthy lifestyles in general population in primary care: randomized controlled trial (evident ii study). JMIR Mhealth Uhealth.

[30] Edney S, Ryan JC, Olds T, Monroe C, Fraysse F, Vandelanotte C, Plotnikoff R, Curtis R, Maher C (2019). User engagement and attrition in an app-based physical activity intervention: secondary analysis of a randomized controlled trial. J Med Internet Res.

[31] Bradway M, Pfuhl G, Joakimsen R, Ribu L, Grøttland A, Årsand E (2018). Analysing mHealth usage logs in RCTs: explaining participants' interactions with type 2 diabetes self-management tools. PLoS One.

[32] Agarwal P, Mukerji G, Desveaux L, Ivers NM, Bhattacharyya O, Hensel JM, Shaw J, Bouck Z, Jamieson T, Onabajo N, Cooper M, Marani H, Jeffs L, Bhatia RS (2019). Mobile app for improved self-management of type 2 diabetes: multicenter pragmatic randomized controlled trial. JMIR Mhealth Uhealth.

[33] Kim H, Faw M, Michaelides A (2017). Mobile but connected: harnessing the power of self-efficacy and group support for weight loss success through mHealth intervention. J Health Commun.

[34] Kim H, Ray CD, Veluscek AM (2017). Complementary support from facilitators and peers for promoting mHealth engagement and weight loss. J Health Commun.

[35] Serrano KJ, Yu M, Coa KI, Collins LM, Atienza AA (2016). Mining health app data to find more and less successful weight loss subgroups. J Med Internet Res.

[36] Vehi J, Regincós Isern J, Parcerisas A, Calm R, Contreras I (2019). Impact of use frequency of a mobile diabetes management app on blood glucose control: evaluation study. JMIR Mhealth Uhealth.

[37] Mitchell M, White L, Lau E, Leahey T, Adams MA, Faulkner G (2018). Evaluating the carrot rewards app, a population-level incentive-based intervention promoting step counts across two Canadian provinces: quasi-experimental study. JMIR Mhealth Uhealth.

[38] Tong HL, Coiera E, Tong W, Wang Y, Quiroz JC, Martin P, Laranjo L (2019). Efficacy of a mobile social networking intervention in promoting physical activity: quasi-experimental study. JMIR Mhealth Uhealth.

[39] Koot D, Goh PS, Lim RS, Tian Y, Yau TY, Tan NC, Finkelstein EA (2019). A mobile lifestyle management program (GlycoLeap) for people with type 2 diabetes: single-arm feasibility study. JMIR Mhealth Uhealth.

[40] Semper HM, Povey R, Clark-Carter D (2016). A systematic review of the effectiveness of smartphone applications that encourage dietary self-regulatory strategies for weight loss in overweight and obese adults. Obes Rev.

[41] Schubart JR, Stuckey HL, Ganeshamoorthy A, Sciamanna CN (2011). Chronic health conditions and internet behavioral interventions: a review of factors to enhance user engagement. Comput Inform Nurs.

[42] Alkhaldi G, Hamilton FL, Lau R, Webster R, Michie S, Murray E (2016). The effectiveness of prompts to promote engagement with digital interventions: a systematic review. J Med Internet Res.

[43] O'Brien HL, Toms EG (2009). The development and evaluation of a survey to measure user engagement. J Am Soc Inf Sci.

[44] Lefebvre C, Tada Y, Hilfiker S, Baur C (2010). The assessment of user engagement with eHealth content: the eHealth engagement scale. J Comput Commun.

