# Supplementary material for: User Engagement With Smartphone Apps and Cardiovascular Disease Risk Factor Outcomes: Systematic Review
Source: JMIR Cardio. 2021 Feb 3;5(1):e18834. doi: 10.2196/18834 (PMC8411427; doi:10.2196/18834)
Supplement: Multimedia Appendix 1 [file cardio_v5i1e18834_app1.docx]

**Multimedia Appendices**

**Multimedia Appendix 1.** Search Strategy

*PubMed, 2007-2018*

(Disease Management [mh] OR "disease management" OR "disease prevention" OR obesity[mh] OR overweight[mh] OR weight loss[mh] OR overweight*[tw] OR over weight*[tw] OR "weight reduction" OR "weight loss maintenance" OR "weight loss" OR "weight control" OR "body weight maintenance" OR "weight regain" OR "body weight changes" OR "weight reduction programs" OR "weight reduction diets" OR obes* [tiab] OR "Heart Diseases"[Mesh] OR "Vascular Diseases"[Mesh] OR "heart disease" [tiab] OR "heart diseases" [tiab] OR "cardiovascular diseases" [tiab] OR "cardiovascular disease" [tiab] OR "coronary artery disease" [tiab] OR "coronary artery diseases" [tiab] OR "heart failure" [tiab] OR hypertens* [tiab] OR "high blood pressure" [tiab] OR "Diabetes Mellitus"[Mesh] OR "Exercise"[Mesh] OR diabete* [tiab] OR exercis* [tiab] OR "physical activity" [tiab])

AND

("Mobile Applications"[Mesh] OR "Computers, Handheld"[Mesh] OR iphone* OR android* OR "smart phone" OR "smart phones" OR "smartphone" OR "mobile app" OR "mobile apps" OR "mHealth" OR "mobile health")

AND

("Randomized Controlled Trial" [Publication Type] OR "Randomized Controlled Trials as Topic"[Mesh] OR "Non-Randomized Controlled Trials as Topic"[Mesh] OR "Evaluation Studies as Topic"[Mesh] OR "random allocation" [mh] OR random* [tiab] OR "quasi-experimental" [tiab] OR "mixed-methods" [tiab] OR "correlation study" OR "correlation studies" OR correlat* [tiab])

AND

(engag* OR experienc* [tiab] OR usage [tiab] OR usability [tiab] OR involv* [tiab])

Limited to 01/01/2007 to 01/20/2018, English

413 citations

*PubMed, 2018-2020*

(Disease Management [mh] OR "disease management" OR "disease prevention" OR obesity[mh] OR overweight[mh] OR weight loss[mh] OR overweight*[tw] OR over weight*[tw] OR "weight reduction" OR "weight loss maintenance" OR "weight loss" OR "weight control" OR "body weight maintenance" OR "weight regain" OR "body weight changes" OR "weight reduction programs" OR "weight reduction diets" OR obes* [tiab] OR "Heart Diseases"[Mesh] OR "Vascular Diseases"[Mesh] OR "heart disease" [tiab] OR "heart diseases" [tiab] OR "cardiovascular diseases" [tiab] OR "cardiovascular disease" [tiab] OR "coronary artery disease" [tiab] OR "coronary artery diseases" [tiab] OR "heart failure" [tiab] OR hypertens* [tiab] OR "high blood pressure" [tiab] OR "Diabetes Mellitus"[Mesh] OR "Exercise"[Mesh] OR diabete* [tiab] OR exercis* [tiab] OR "physical activity" [tiab])

AND

("Mobile Applications"[Mesh] OR "Computers, Handheld"[Mesh] OR iphone* OR android* OR "smart phone" OR "smart phones" OR "smartphone" OR "mobile app" OR "mobile apps" OR "mHealth" OR "mobile health")

AND

("Randomized Controlled Trial" [Publication Type] OR "Randomized Controlled Trials as Topic"[Mesh] OR "Non-Randomized Controlled Trials as Topic"[Mesh] OR "Evaluation Studies as Topic"[Mesh] OR "random allocation" [mh] OR random* [tiab] OR "quasi-experimental" [tiab] OR "mixed-methods" [tiab] OR "correlation study" OR "correlation studies" OR correlat* [tiab])

AND

(engag* OR experienc* [tiab] OR usage [tiab] OR usability [tiab] OR involv* [tiab])

Limited to 01/20/2018 to 01/20/2020, English

380 citations

*Embase, 2007-2018*

| No. | Query | Results |
| --- | --- | --- |
| #8 | #6 NOT #7 | 518 |
| #7 | 'conference paper' OR conference:it | 3,630,326 |
| #6 | #1 AND #2 AND #3 AND #4 AND [english]/lim AND [2007-2018]/py | 817 |
| #5 | #1 AND #2 AND #3 AND #4 | 842 |
| #4 | engag*:ti,ab OR experienc*:ti,ab OR usage:ti,ab OR usability:ti,ab OR involv*:ti,ab | 3,944,050 |
| #3 | 'randomized controlled trial'/exp OR 'evaluation study'/exp OR 'randomization'/exp OR 'quasi experimental study'/exp OR 'correlational study'/exp OR random*:ti,ab OR nonrandom*:ti,ab OR 'quasi-experimental':ti,ab OR 'mixed methods':ti,ab OR ((correlat* NEAR/3 stud*):ti,ab) OR correlat*:ti,ab | 3,619,744 |
| #2 | 'mobile application'/exp OR 'personal digital assistant'/exp OR 'mobile phone'/exp OR iphone*:ti,ab OR android*:ti,ab OR ((smart NEAR/3 phone*):ti,ab) OR smartphone*:ti,ab OR ((mobile NEAR/3 app*):ti,ab) OR mhealth:ti,ab OR ((mobile NEAR/3 health):ti,ab) | 29,589 |
| #1 | 'disease management'/exp OR 'obesity'/exp OR 'body weight loss'/exp OR 'body weight control'/exp OR 'heart disease'/exp OR 'vascular disease'/exp OR 'hypertension'/exp OR 'diabetes mellitus'/exp OR 'exercise'/exp OR 'physical activity'/exp OR ((disease NEAR/3 (mangement OR prevention)):ti,ab) OR obes*:ti,ab OR overweight:ti,ab OR ((weight NEAR/3 (loss OR reduction OR control OR maintenance OR regain OR changes)):ti,ab) OR (((heart OR cardiovascular OR vascular OR coronary) NEAR/3 (disease* OR failure)):ti,ab) OR hypertens*:ti,ab OR diabet*:ti,ab OR exercis*:ti,ab OR 'high blood pressure':ti,ab OR 'physical activity':ti,ab | 7,209,857 |

*Embase, 2018-2020*

| No. | Query | Results |
| --- | --- | --- |
| #8 | #6 NOT #7 | 449 |
| #7 | 'conference paper' OR conference:it | 4,437,874 |
| #6 | #1 AND #2 AND #3 AND #4 AND [english]/lim AND [2018-2020]/py | 665 |
| #5 | #1 AND #2 AND #3 AND #4 | 1,554 |
| #4 | engag*:ti,ab OR experienc*:ti,ab OR usage:ti,ab OR usability:ti,ab OR involv*:ti,ab | 4,552,822 |
| #3 | 'randomized controlled trial'/exp OR 'evaluation study'/exp OR 'randomization'/exp OR 'quasi experimental study'/exp OR 'correlational study'/exp OR random*:ti,ab OR nonrandom*:ti,ab OR 'quasi-experimental':ti,ab OR 'mixed methods':ti,ab OR ((correlat* NEAR/3 stud*):ti,ab) OR correlat*:ti,ab | 4,201,915 |
| #2 | 'mobile application'/exp OR 'personal digital assistant'/exp OR 'mobile phone'/exp OR iphone*:ti,ab OR android*:ti,ab OR ((smart NEAR/3 phone*):ti,ab) OR smartphone*:ti,ab OR ((mobile NEAR/3 app*):ti,ab) OR mhealth:ti,ab OR ((mobile NEAR/3 health):ti,ab) | 45,492 |
| #1 | 'disease management'/exp OR 'obesity'/exp OR 'body weight loss'/exp OR 'body weight control'/exp OR 'heart disease'/exp OR 'vascular disease'/exp OR 'hypertension'/exp OR 'diabetes mellitus'/exp OR 'exercise'/exp OR 'physical activity'/exp OR ((disease NEAR/3 (mangement OR prevention)):ti,ab) OR obes*:ti,ab OR overweight:ti,ab OR ((weight NEAR/3 (loss OR reduction OR control OR maintenance OR regain OR changes)):ti,ab) OR (((heart OR cardiovascular OR vascular OR coronary) NEAR/3 (disease* OR failure)):ti,ab) OR hypertens*:ti,ab OR diabet*:ti,ab OR exercis*:ti,ab OR 'high blood pressure':ti,ab OR 'physical activity':ti,ab | 8,240,396 |

*CINAHL, 2007-2018*

| # | Query | Limiters/ Expanders | Last Run Via | Results |
| --- | --- | --- | --- | --- |
| S7 | S1 AND S2 AND S3 AND S4 | Limiters - Published Date: 20070101-20181231  Narrow by Language: - english  Search modes - Boolean/Phrase | Interface - EBSCOhost Research Databases  Search Screen - Advanced Search  Database - CINAHL Plus with Full Text | 136 |
| S6 | S1 AND S2 AND S3 AND S4 | Limiters - Published Date: 20070101-20181231  Search modes - Boolean/Phrase | Interface - EBSCOhost Research Databases  Search Screen - Advanced Search  Database - CINAHL Plus with Full Text | 136 |
| S5 | S1 AND S2 AND S3 AND S4 | Search modes - Boolean/Phrase | Interface - EBSCOhost Research Databases  Search Screen - Advanced Search  Database - CINAHL Plus with Full Text | 141 |
| S4 | (MH "Usability Study") OR ( engag* OR experienc* OR usage OR usability OR involv* ) | Search modes - Boolean/Phrase | Interface - EBSCOhost Research Databases  Search Screen - Advanced Search  Database - CINAHL Plus with Full Text | 540,664 |
| S3 | ( (MH "Randomized Controlled Trials") OR (MH "Evaluation Research") OR (MH "Random Assignment") OR (MH "Quasi-Experimental Studies+") OR (MH "Nonrandomized Trials") OR (MH "Correlational Studies") ) OR ( random* OR "quasi-experimental" OR "mixed-methods" OR (correlat* N3 stud*) ) | Search modes - Boolean/Phrase | Interface - EBSCOhost Research Databases  Search Screen - Advanced Search  Database - CINAHL Plus with Full Text | 393,247 |
| S2 | ( (MH "Mobile Applications") OR (MH "Computers, Hand-Held+") OR (MH "Smartphone") ) OR ( iphone* OR android* OR (smart N3 phone*) OR (mobile N3 app*) OR smartphone* OR (mobile N3 health) ) | Search modes - Boolean/Phrase | Interface - EBSCOhost Research Databases  Search Screen - Advanced Search  Database - CINAHL Plus with Full Text | 12,759 |
| S1 | ( (MH "Disease Management+") OR (MH "Obesity+") OR (MH "Weight Loss+") OR (MH "Heart Diseases+") OR (MH "Vascular Diseases+") OR (MH "Diabetes Mellitus") OR (MH "Diabetes Mellitus, Type 2") OR (MH "Hypertension+") OR (MH "Exercise+") OR (MH "Physical Activity") ) OR ( (disease N3 (management OR prevention)) OR obes* OR overweight OR (weight N3 (loss OR regain OR control OR changes OR maintenance OR reduction)) OR obes* ) OR ( heart or cardiac or cardiovascular OR coronary) N3 (disease OR failure)) OR hypertens* OR "high blood pressure" OR diabet* OR exercis* ) | Search modes - Boolean/Phrase | Interface - EBSCOhost Research Databases  Search Screen - Advanced Search  Database - CINAHL Plus with Full Text | 971,695 |

*CINAHL, 2018-2020*

| # | Query | Limiters/ Expanders | Last Run Via | Results |
| --- | --- | --- | --- | --- |
| S7 | S1 AND S2 AND S3 AND S4 | Limiters - Published Date: 20181201-20200131  Narrow by Language: - english  Search modes - Boolean/Phrase | Interface - EBSCOhost Research Databases  Search Screen - Advanced Search  Database - CINAHL Plus with Full Text | 68 |
| S6 | S1 AND S2 AND S3 AND S4 | Limiters - Published Date: 20181201-20200131  Search modes - Boolean/Phrase | Interface - EBSCOhost Research Databases  Search Screen - Advanced Search  Database - CINAHL Plus with Full Text | 68 |
| S5 | S1 AND S2 AND S3 AND S4 | Search modes - Boolean/Phrase | Interface - EBSCOhost Research Databases  Search Screen - Advanced Search  Database - CINAHL Plus with Full Text | 272 |
| S4 | (MH "Usability Study") OR ( engag* OR experienc* OR usage OR usability OR involv* ) | Search modes - Boolean/Phrase | Interface - EBSCOhost Research Databases  Search Screen - Advanced Search  Database - CINAHL Plus with Full Text | 710,477 |
| S3 | ( (MH "Randomized Controlled Trials") OR (MH "Evaluation Research") OR (MH "Random Assignment") OR (MH "Quasi-Experimental Studies+") OR (MH "Nonrandomized Trials") OR (MH "Correlational Studies") ) OR ( random* OR "quasi-experimental" OR "mixed-methods" OR (correlat* N3 stud*) ) | Search modes - Boolean/Phrase | Interface - EBSCOhost Research Databases  Search Screen - Advanced Search  Database - CINAHL Plus with Full Text | 547,474 |
| S2 | ( (MH "Mobile Applications") OR (MH "Computers, Hand-Held+") OR (MH "Smartphone") ) OR ( iphone* OR android* OR (smart N3 phone*) OR (mobile N3 app*) OR smartphone* OR (mobile N3 health) ) | Search modes - Boolean/Phrase | Interface - EBSCOhost Research Databases  Search Screen - Advanced Search  Database - CINAHL Plus with Full Text | 19,833 |
| S1 | ( (MH "Disease Management+") OR (MH "Obesity+") OR (MH "Weight Loss+") OR (MH "Heart Diseases+") OR (MH "Vascular Diseases+") OR (MH "Diabetes Mellitus") OR (MH "Diabetes Mellitus, Type 2") OR (MH "Hypertension+") OR (MH "Exercise+") OR (MH "Physical Activity") ) OR ( (disease N3 (management OR prevention)) OR obes* OR overweight OR (weight N3 (loss OR regain OR control OR changes OR maintenance OR reduction)) OR obes* ) OR ( heart or cardiac or cardiovascular OR coronary) N3 (disease OR failure)) OR hypertens* OR "high blood pressure" OR diabet* OR exercis* ) | Search modes - Boolean/Phrase | Interface - EBSCOhost Research Databases  Search Screen - Advanced Search  Database - CINAHL Plus with Full Text | 1,105,290 |
