# Supplementary material for: User Engagement With Smartphone Apps and Cardiovascular Disease Risk Factor Outcomes: Systematic Review
Source: JMIR Cardio. 2021 Feb 3;5(1):e18834. doi: 10.2196/18834 (PMC8411427; doi:10.2196/18834)
Supplement: Multimedia Appendix 2 [file cardio_v5i1e18834_app2.docx]

**Multimedia Appendices**

**Multimedia Appendix 2.** Study Characteristics

| Author (Year), Country, Study design | Sample characteristics & Setting | Intervention description | Measures of user engagement w/ smartphone apps & Main outcome | Results: Relationship between user engagement w/ smartphone apps & main outcome |
| --- | --- | --- | --- | --- |
| Change in Anthropometrics | | | | |
| Bennett et al. (2018),^23^  USA  RCT  12 mo intervention duration; 6 & 12 mo follow-ups | 351 patients w/ obesity & dx of HTN, DM, or hyperlipidemia enrolled via primary care EHR ^†^(CS)  Age: ^§^50.7 (8.9)  Sex: 68% male  BMI: 35.9 (3.9) Race: 52% Black | I: App for self-monitoring of behavior change goals w/ tailored feedback, smart scale, dietitian-delivered counseling calls, & clinician counseling at regular medical visits informed by app data.  C: Usual care offered by clinician, self-help info., list of community resources for weight management, & newsletter. | User engagement at 12 mos:  (1) ≥ 80% of expected self-monitoring  (2) ≥ 80% receipt of counseling calls  (3) ≥ 5 days/wk of self-weighing  (4) meeting criteria 1-3  Main outcome: Weight | (1) ≥ 80% of expected self-monitoring had -3.5kg ^‡^(-5.9, -1.2) more weight loss than < 80% (***P*=.004**)  (2) ≥ 80% receipt of counseling calls had -3.0 kg (-5.3, -0.7) more weight loss than < 80% (***P*=.01**)  (3) ≥ 5 days/wk of self-weighing had -4.4kg (-6.8, -2.0) more weight loss than <5 days (***P*=.0004**)  (4) Those who met all previous criteria had -4.5kg (-7.0, -2.1) more weight loss than those who didn’t (***P*=.0003**) |
| Godino et al. (2016),^17^  USA  RCT  24 mo intervention duration; 6, 12, 18, & 24-mo follow-ups | 404 college students w/ overweight/ obesity enrolled on college campuses (CS)  Age: 22.7 (3.8)  Sex: 70% female  BMI: 28.5 (27.9, 29.0)  Race: 42% White | I: Social Mobile Approaches to Reduce Weight w/ PA/diet goals, tracking, & feedback via integrated Facebook, 3 apps, real-time location-based SMS, emails, blog posts, & health coach.  C: Diff. website on weight loss & email newsletters. | User engagement: Sum of recorded Facebook (posts, likes, comments) & app (steps/day entry) interactions, texts sent & answered, & contact w/ health coach. High vs. low engagement decided by median split.  Main outcome: Weight | High engagement did not result in greater weight loss than low engagement, *P*>.05 at all time points (actual data not available). |
| Johnston et al. (2013),^19^ USA  RCT  6 mo intervention duration; 3 & 6-mo follow-ups | 292 adult volunteers w/ overweight/ obesity enrolled in community (CS)  Age: 46.5 (10.5) Sex: 90% female  BMI: 33.0 (3.6)  Race: 91% White | I: Weight Watchers (WW) app/website w/ food/PA plan, group support, & behavior change skills. WW staff held meetings on eTools (education, self-monitoring, recipes).  C: Publicly available print materials on diet/PA & other resources. | User engagement: Self-reported wkly use at 3 & 6 mos:  (1) High frequency users (HFU): using≥2x/wk  (2) Low frequency users (LFU): using≤1x/wk  Main outcome: Weight | 3-mos: HFU 8.3x more likely to reach 5% weight loss compared to LFU (***P*<.001**)  6-mos (ref: LFU): More usage correlated w/ greater weight loss (r=0.28, ***P*<.01**); HFU 2.0x (1.0, 4.1) more likely to reach 5% weight loss (***P*<.05**), & 3.3x (1.3, 8.1) more likely to reach 10% weight loss (***P*<.05**). Among those w/ ≥10% weight loss, 31.8% were HFU & 12.5% LFU. |
| Kim et al. (2017a),^33^  USA  Retrospective study  Examined 6-mo period | 384 adult Noom users selected via random sampling  Age: 34.2 (10.6) Sex: 80% female  BMI: 30.6 (6.54)  Race: no data | Noom weight loss commercial app. Users signed up into groups of 6-12 users plus facilitator. Recommended calorie intake & fitness/nutrition articles. Could interact w/ others in their group by commenting on other users’ posts or liking their posts. | User engagement:  (1) Logged meals/snacks  (2) Original posts (status updates, food diaries/ photos, goals), & response to others’ posts (comments & likes)  Main outcome: BMI | (1) Numbers of food logs was **stat. sig.** associated w/ weight loss success **(0.37, no p-value)**  (2) Group participation (original posts & response to others’ posts) was **stat. sig.** associated w/ weight loss success **(0.22, no p-value)** |
| Kim et al. (2017b),^34^  USA  Retrospective study  Examined 6-mo period | 301 adult Noom users selected via random sampling  Age: 34.7 (10.8) Sex: 73% female  BMI: 32.3 (5.98)  Race: no data | Noom weight loss commercial app. Users signed up into groups of 6-12 users plus facilitator. Recommended calorie intake & fitness/nutrition articles. Could interact w/ others in their group by commenting on other users’ posts or liking their posts. | User engagement:  (1) Articles marked as read  (2) Original posts  (3) Response to others’ posts  Main outcome: Weight | (1) Numbers of articles read was **stat. sig.** associated w/ weight loss success **(0.18, no p-value)**  (2) Number of original posts was **stat. sig.** associated w/ weight loss success **(0.28, no p-value)**  (3) Leaving responses was not stat. sig. associated w/ weight loss success (data not available) |
| Lin et al. (2018),^24^  USA  RCT  24 mo intervention duration; 6, 12, & 24 mos follow-ups | 242 adults (I^1^ + I^2^) w/ overweight/ obesity enrolled in community (CS)  Age: 29.3 (4.2)  Sex: 70% female  BMI: 35.3 (7.9)  Race: 55% White | I^1^: CITY app for self- monitoring weight, diet/calories & PA; social support; feedback; goal setting, challenges, prompting; & rewards. Bluetooth scale.  I^2^: 6 wkly 2-hr group dietitian led sessions, phone call/mo for 22 mos. Access to CITY w/o reminders/prompts.  C: Handouts on healthy lifestyle. | User engagement:  (1) mean % of days any app component was used  (2) mean number of times any app component was used/day  (3) mean % of days participants self-weighed  Main outcome: Weight | 0-6 mos: (1) mean % of the days any app component was used (I^1^: ***P*=.04,** r= −.213; I^2^: ***P*=.004**, r= −.319), (2) mean number of times any app component was used/day (I^1^: ***P*=.006**, r= −.264; I^2^: ***P*=.001**, r= −.308), & (3) mean % of days participants self-weighed (I^1^: ***P*=.01**, r= −.297; I^2^: ***P*=.003**, r= −.354) were each correlated w/ weight change  7-12 mos: (1) mean % of the days any app component was used (I^1^: *P*=.68, r= .004; I^2^: ***P*=.02**, r= −.124), (2) mean number of times any app component was used/day (I^1^: *P*=.70, r= −.035; I^2^: ***P*=.02**, r= −.109), & (3) mean % of days participants self-weighed (I^1^: *P*=.46, r= −.031; I^2^: ***P*=.05**, r= −.139), where engagement with I^2^ remained correlated w/ weight change but I^1^ did not.  13-24 mos: stat. sig. associations did not persist for either I^1^ or I^2^. |
| Patel et al. (2019a),^25^  USA  Patel et al. (2019b),^26^ USA  RCT  3 mo intervention duration; 1, 3, & 6 mos follow-ups | 100 adults w/ overweight/ obesity enrolled in community (CS)  Age: 42.7 (11.7)  Sex: 84% female  BMI: 31.9 (4.5)  Race: 67% White | I^1^: MyFitnessPal for self- monitoring diet; weight loss/ calorie goals; reminders; & progress graphs. No structured dietary advice.  I^2^: I^1^ + daily weight tracking. Wkly email feedback on weight loss/calorie goals, weight & diet tracking, & training on app & nutrition/behavior change.  I^3^: I^1^ + I^2^ but diet tracking & calorie goal feedback ≥ wk 5. | User engagement: % of days entries made for weight (for I^2^ and I^3^ only, I^1^ not asked to complete weight tracking) & diet (for I^1^ and I^2^ only, I^3^ not asked to track diet for entire study period) [only counted days w/ ≥800 kcal/day recorded]; % of action plans completed (for I^2^ and I^3^ only, I^1^ not asked to complete action plans)  Main outcome: Weight | Early responders (≥2% weight loss at 1 mo) completed more action plans than early non‐responders (<2% weight loss; ***P*=.02**) & self‐monitored weight & diet more frequently at all time intervals (***P*s<.01**)  % of days weight tracked was stat. sig. associated w/ 3 mo weight change in I^2^ (rs=−.48, ***P*=.02**) & I^3^ (rs=−.47, ***P*=.01**). I^1^ not included in analysis bc not instructed to track weight.  % of days w/ diet entries was stat. sig associated w/ 3 mo weight change in I^1^ (rs=−.58, ***P*=.003**) but not in I^2^ (rs=−.25, *P*=.24). The % of days diet was tracked starting in wk 5 for I^3^ was stat. sig. associated w/ weight change at 3 mos (rs=−.44, ***P*=.02**) |
| Serrano et al. (2016),^35^  USA  Retrospective cross-sectional correlational  study | 324,649 adults w/ overweight/ obesity w/ app from 2008-2014 selected via CS  Age: 36.3 (11.8)  Sex: 70% female  BMI: 33.5 (6.7)  Race: no data | LoseIt! weight loss iOS, Android, & web app; tracking tools (barcode scanners); connection w/ Fitbit, RunKeeper, & friends; & feedback. Personalized calorie plan + logging diet, exercise, & weight through self-report or devices. | User engagement: Number of app customizations and food & weight entries logged:  (1) Occasional users: Weighed in <6.5x  (2) Basic users: Weighed in ≥6.5x w/ <40 food days logged  (3) Power users: Weighed in ≥6.5x w/ ≥40 food days logged  Main outcome: 5% weight loss success | 4.87% occasional, 37.61% basic, & 72.7% power users achieved 5% weight loss  Odds of achieving 5% weight loss by number of customizations:  1 customization: **OR=5.27 (5.11,5.44)**  2 customizations: **OR=12.39 (11.99, 12.81)**  3 customizations: **OR=22.42 (21.56, 23.31)**  4 customizations: **OR=48.30 (46.23, 50.46)** |
| Tanaka et al. (2018),^27^  Japan  RCT  8 wk intervention duration; 8 wk follow-ups | 112 Japanese adults w/ ≥1 cardiometabolic risk factor enrolled from Tokyo companies (CS)  Age: 46.7 (9.8)  Sex: 98% male  BMI: 28.1 (3.2) | I: Wellness Coach weight loss program delivered by app w/ self-monitoring, nutrition professional feedback, social support, & individually tailored program. Told to measure weight 2x/day & upload meal photos 3x/day.  C: No intervention but offered app after 12-wk study period. | User engagement: total number of meal photo uploads divided into tertiles over 8 wks:  (1) <29 uploads  (2) 29-127 uploads  (3) ≥128 uploads  Main outcomes: Weight, waist circumference, HbA1c | Stat. sig. associations btw more frequent upload of meal photos & changes in weight, waist circumference, & HbA1c (***P*<.05** for all).  Weight: -1.9 kg (**0.6, 3.2**) for 2^nd^ tertile & -3.5 kg (**2.2, 4.8**) for 3^rd^ tertile, as compared to the 1^st^ tertile.  Waist circumference: -3.2 cm (**1.4, 4.9**) for 2^nd^ tertile & -3.9 cm (**2.1, 5.7**) for 3^rd^ tertile.  HbA1c: -0.09% (−0.28, 0.11) for 2^nd^ tertile & -0.26% (**−0.46, −0.07**) for 3^rd^ tertile. |
| van Beurden et al. (2019),^28^ United Kingdom  RCT  3 mo intervention duration; 1 & 3 mo follow-ups | 88 participants w/ overweight/ obesity, ≥ 16 yrs enrolled in community (CS)  Age: 46.8 (13.9)  Sex: 65% female  BMI: 33.3 (6.1)  Race: 95% White | I: ImpulsePal app for reducing unplanned & unhealthy snacking, drinking, & overeating + manage impulses. Gamification & personalization. Encouraged to use the app for the first 4 wks but allowed to use for the whole study period.  C: ImpulsePal app after 3 mos. | User engagement: total time spent using the app & number of days the app had been accessed.  Main outcomes: Weight | Total minutes using the app was not sig. correlated w/ weight loss at 1 mo (r= –0.16) or at 3 mos (r= 0.04).  Number of days using the app was not sig. correlated w/ weight loss at 1 mo (r= –0.01) or at 3 mos (r= –0.02). |
| Change in Health Behavior | | | | |
| Duncan et al. (2014),^16^ Australia  RCT  9 mo intervention duration; 3 & 9-mo follow-ups | 317 adult men enrolled in community (CS)  Age: 44(SE:0.80)  Sex: 100% male  BMI: 89% obese/ overweight  Race: no data | I: C + ManUp app (self-monitor behavior, weight, & challenges) & website (monitor friends’, challenges, PA/diet education, & anthropometrics).  C: Printed PA/diet education, challenges, & self-monitoring. | User engagement (app & website combined):  (1) Log-ins/wk  (2) Self-monitoring entries/wk  Main outcomes: Self-reported PA (Active Australia survey) & dietary behavior score, & high-fiber bread & low-fat milk intake | PA mins/wk: *logins/wk:* 1.0 (0.98,1.01) at 3 mos, *P*=.43; 1.0 (0.99,1.00) at 9 mos, *P*=.25; *Self-monitoring/wk:* 1.00 (0.997, 1.01) at 3 mos, *P*=.38; 1.00 (1.00-1.01) at 9 mos, *P*=.10.  PA sessions/wk: *logins/wk:* 0.99 (0.98,1.01) at 3 mos, *P*=.19; 1.00 (0.99,1.00) at 9 mos, *P*=.41; *Self-monitoring/wk:* 1.01 (1.00-1.01) at 3 mos, P=.05; 1.00 (1.00-1.01) at 9 mos, *P*=.16  Dietary score: *logins/wk:* 1.00 (1.00-1.00) at 3 mos, *P*=.65; 1.00 (1.00-1.00) at 9 mos, *P*=.11; *Self-monitoring/wk:* 1.00 (0.99-1.00) at 3 mos, *P*=.76; 1.00 (1.00-1.00) at 9 mos, *P*=.63  No stat. sig. associations found btw measures of user engagement & high-fiber bread & low-fat milk intake. |
| Edney et al. (2019),^30^ Australia  RCT  100 day intervention duration; 3 mo follow-up | 301 adults enrolled in community (CS)  Age: 42 (12)  Sex: 74% female  BMI: 79% obese/ overweight  Race: no data | I^1^: Active Team gamified app encouraged 10,000 steps/day. Social & gamified features (leaderboard, gifts/medals, newsfeed, & PA challenges) designed to encourage app use & in-app interaction w/ friends. Self-monitoring of steps & push notifications. Pedometer & wkly email progress updates.  I^2^: Simplified Active Team app w/o gamified or social features. Self-monitoring of steps & push notifications. Pedometer & wkly email progress updates.  C: Waitlist control group. | User engagement: number of times app features were used (step calendar; newsfeed; challenge, gift, & friends pages). Daily active users: number of I^1^ & I^2^ who accessed app every day. Super users: whose total app use fell into the top quartile of all users.  Main outcome: Step count & self-reported PA | Super users were more likely to be a gamified rather than basic app user (χ21=29.4; **P<.001**)  Super users had greater increases in step count at 3 mos (t239=4.1; ***P*<.001**) than regular users. No diff. btw super & regular users on self-reported PA at 3 mos.  Weak, sig. total app use-by-time interaction effect for step count (F1,272=4.5; ***P*=.04**) & self-reported PA (F1,304=6.56; ***P*=.01**), where higher total app use was associated w/ greater increases in PA at 3-mos.  Sig. group by time interaction, super users completed 28.2 (9.4, 46.9) more mins of objective MVPA than regular users (F1,272=4.76; ***P*=.03**).  Differences btw super & regular users for self-reported MVPA favored super users (mean 89.7, SE 43.4) but did not reach stat. sig. (F1,297=3.31; *P*=.07). |
| Garcia- Ortiz et al. (2018),^29^  Spain  RCT  3 mo intervention duration; 3 & 12 mo follow-ups | 833 participants <70 yrs selected via random sampling from 6 primary care centers from Evident I study  Age: 51.9 (12.1)  Sex: 62% female  BMI: 27.9 (4.9)  Race: no data | I: C + app for self-monitoring of diet & PA. PA tracking w/ ActiGraph GT3X & manual entry. Generated reports on diet & calorie consumption, & PA. Suggestions to improve eating habits & increase PA toward targeted 10,000 steps/day. 15 min training on device use w/ follow up at 1 wk.  C: 30-min counseling on PA & Mediterranean diet. | User engagement: number of days in app over 3 mos:  (1) 0 days  (2) 1-30 days  (3) 31-60 days  (4) 61-90 days  Main outcomes: Mediterranean diet adherence score, MVPA (ActiGraph), and PA & MET mins/wk (PA Recall survey) | 12 mos: No correlation btw number of days the app was used & change in Mediterranean diet adherence score or PA Recall survey. Stat. sig. correlation btw steps/day (ρ=.114, ***P*=.046**), mins of moderate activity (ρ=.112, ***P*=.049**), mins of MVPA (ρ=.113, ***P*=.047**) & number of days of app use. |
| Mitchell et al. (2018),^37^ Canada  Single group pre-and-post quasi-exp. study  3 mo intervention duration; 3 mo follow-up | 32,229 adults enrolled in community (CS)  Age: 33.7 (11.6)  Sex: 66% female  BMI: no data  Race: no data | The Carrot Rewards app aims to engage users in healthy behaviors, such as walking, through goal setting, graded tasks, biofeedback, small incentives tied to a daily step count goal & self- monitoring. | User engagement: days of app use. Dichotomized into high or low engagers based on median percentage of days when a PA challenge was accepted.  Main outcome: Step count | Low app engagers had a 490.75 (**-551.21, -428.29**) reduction in mean daily steps at 12-wks.  High app engagers had a 630.90 (**575.43, 686.36**) increase in mean daily step count at 12-wks.  Engagement showed a significant moderating effect on the intervention outcome in all models (***P*<.001**). |
| Tong et al. (2019),^38^ Australia  Single group pre-and-post quasi-exp. study  6 mo intervention duration; 6 mo follow-up | 55 university students & staff selected via purposive sampling  Age: 23.6 (4.6)  Sex: 51% female  BMI: 26.6 (6.8)  Race: no data | App, Fitbit wearable tracker, SMS messages, & emails. App w/ self-monitoring of PA, social support, & social comparison. Prompts & cues via emails & SMS sent every 2 wks. | User engagement: mean number of days of usage, median was used as a cut point to determine frequent vs infrequent usage  Main outcome: Step count | No stat. sig. changes in average daily step count btw frequent (n=28) & infrequent (n=27) app users (*P*=.42) or btw frequent & infrequent users of social features (*P*=.25).  Total engagement w/ app was not associated w/ change in daily step counts (Kendall tau-b=–0.11, *P*=.25). |
| Wang et al. (2016),^22^  USA  RCT  6 wk intervention duration; 6-wk follow-up | 67 adult volunteers w/ overweight/ obesity & low PA enrolled in community (CS)  Age: 48.2 (11.7)  Sex: 91% female  BMI: 31.0 (3.7)  Race: 67% White | I^1^: I^2^ + PA texts 3x/day per participant’s preference.  I^2^: Fitbit One for self-monitoring w/ tracker, website, & app. Tracker gives PA feedback & ability to upload data to website or app for daily PA summaries. | User engagement: Self-reported app use versus no use. For app users: 6-point scale to assess use frequency  Main outcome: Step count | App users had +545 (265) steps/wk vs. non-users who had −28 (264) steps/wk, *P*=.12; Among app users steps/wk increased by 545 (265) from baseline to 6 wks, ***P*=.04.** |
| Change in Risk Factors/Biomarkers | | | | |
| Agarwal et al. (2019),^32^ Canada  RCT  6 mo intervention duration; 3 & 6 mo follow-ups | 223 adults w/ Type 2 DM enrolled from hospital-based DM education programs (CS)  Age: 51.8 (10.7)  Sex: 52% male  BMI: no data  Race: 56% non-White | I: BlueStar app for Type 2 DM w/ self-monitoring of daily BG, exercise, & diet. Customized, real time messages based on self-monitoring input. App generated summary report for user’s clinician.  C: Received usual care from DM education program & PCP for first 3 mos then BlueStar app for 3-6 mos. | User engagement: frequency of use (mean uses/wk) overall & by feature  Main outcomes: HbA1c & patient-reported DM self-care behaviors (Problem Areas in Diabetes scale) | Each additional day of app use corresponded w/ a 0.016- point decrease in 3-mo HbA1c levels (−0.03, −0.003; ***P*=.02**). Thus, 25 days of additional app use corresponded w/ a HbA1c reduction of 0.4%.  Increased use of exercise feature weakly correlated w/ lower HbA1c at 3 mos (ρs=−0.33; ***P*=.01**). Total days w/ at least one app use; carbs, food, & BG tracking; & clinician reports non-stat. sig. weak correlations.  Exploratory analysis assessing association btw app use & patient-reported DM self-care behaviors at 3 mos was not stat. sig. (−0.28, 0.091; *P*=.32). |
| Bradway et al. (2018),^31^ Norway  RCT  12 mo intervention duration; 12-mo follow-up | 151 adults w/ type 2 DM & HbA1c ≥7.1% enrolled in community (CS)  Age: 57.3 (12.0)  Sex: 59% male  BMI: 31.7 (6.0)  Race: no data [38] | I^1^: Few Touch Application (FTA) including app & Bluetooth glucose meter to track & review BG, PA & diet, goals, & general disease info. App training.  I^2^: I^1^ + health counseling by a DM nurse.  C: Usual care regulated by national guidelines, which include ≥1 visit annually w/ general practitioner. [38] | User engagement:  App/meter usage:  (1) Non-users  (2) Short-term users: < 3 continuous mos  (3) Long-term users: ≥3 continuous mos  (1) Registrations: self- monitoring of diet, exercise, or BG.  (2) Navigations: viewing of diet, exercise, or BG entries or disease info.  Main outcome: HbA1c | Non-users, short-term & long-term users differed in change in HbA1c [F(2, 74)=3.794, ***P*=.027**, η2 = .093]. Only diff. was btw the non-users, who increased their HbA1c by 0.33% & the long-term users, who reduced their HbA1c by -0.86%, ***P*=.021**, Cohen’s d = .311.  Change in HbA1c did not correlate w/ the number of registrations or navigations, (all *P*>.15).  Comparing two groups (diet/exercise registrations + navigations vs. BG registrations + navigations & overall navigations), found a non-stat. sig. group diff. in HbA1c (F(1, 55) = 3.642, *P*=.062, η2 = .062). |
| Hartin et al. (2016),^18^  USA  RCT  6 mo intervention duration; 6-mo follow-up | 144 adult volunteers enrolled in community (CS)  Age: 54.6 (6.9)  Sex: 67% female  BMI: ≤41  Race: 97% White | I: iOS & Android Gray Matters Alzheimer’s Disease app: risk & prevention education (food, physical, social, cognitive, sleep, & stress); self-reporting PA, nutrition, mental health; & feedback. PA monitor, coach, booster events, & website (education).  C: No intervention | User engagement:  (1) Logs completed/day  (2) Questions completed/day  Main outcomes: BMI, resting HR, SBP, DBP, total cholesterol, blood carotenoids, triglycerides, BG, & insulin levels | (1) Logs completed/day & outcome: BMI (r =.016, *P*=.87); DBP (r =.064, *P*=.52); SBP (r =.28, *P*=.78); resting HR (r =.28, *P*=.78); blood carotenoids (r = −.105, *P*=.29); triglycerides (r =.145, *P*=.15); BG (r = −.88, *P*=.38); insulin levels (r = −.105, *P*=.30); total cholesterol (r =.145, *P*=.91)  (2) Avg. questions completed/day & outcome: Total cholesterol (r =.228, ***P*=.02**); HDL (t_97.74_=−3.051, ***P*=.003**) |
| Koot et al. (2019),^39^ Singapore  Single group pre-and-post quasi-exp. study  6 mo intervention duration; 6-mo follow-up | 100 adults w/ T2DM enrolled from community healthcare facility (CS)  Age: 53.5 (9.6)  Sex: 50% male  BMI: 29.8 (5.0)  Race: 45% Chinese | GlycoLeap app, glucometer kit (manual input into app), wireless weight scale, & resistance strength band. 24 lessons on DM & self-management. Uploading meal photos w/ health coaches rating healthiness. Print materials on devices & healthy diet/weight loss. | User engagement: total number of logs, messages, or lessons completed. More engaged users if > median & less engaged users if ≤ median.  Main outcomes: HbA1c & weight | HbA1c decreased by an average of 1.0% point more among those who logged their weight more (***P*=.007**). HbA1c did not decrease sig. for those who logged more BG levels, meals, completed more lessons, or had more messages w/ health coaches.  No stat. sig. associations found btw change in weight & engagement (data not provided). |
| Mattila et al. (2013),^20^ Finland  RCT  12 mo intervention duration; 12-mo follow-up | 352 adult city employees w/ elevated health risks enrolled in community (CS)  I^1^ (overall sample info. not given): Age: 44.6 (7.1)  Sex: 71% female  BMI: 27.1 (4.0)  Race: no data | I^1^: I^2^ + devices (pedometer, scale, HR belt), apps (Wellness Diary, Mobile Coach, SelfRelax), & webservices (messaging btw pts & leaders, Nutritioncode, Hyperfit, Wellness Diary Connected)  I^2^: In-person group meetings on weight, diet, PA, sleep, stress, alcohol consumption, smoking.  C: Standard occupational healthcare | User engagement: Number of users (tried tech ≥1x), usage days (day w/ any log), & usage wks (wk ≥1 log).  Study divided into 13 (baseline period & 12 four-wk blocks). Sustained users: used any tech even once on ≥11 of 13 blocks.  Main outcomes: Weight, body fat, BMI, waist circumference, SBP, DBP, total cholesterol, triglycerides & METmax | *Weight change:* Sustained users: -1.2kg (-2.38, -0.01); Non-sustained users: 0.6kg (-0.095,1.27); ***P*=.03**; *Body Fat:* Sustained: -0.9% (-1.64,-.09); Non-sustained: 0.3% (-0.28, 0.73); ***P*=.02**; *Waist:* Sustained: -1.4cm (-2.60, -0.2); Non-sustained: 0.7cm (-0.21,1.66); ***P*=.01**; *BMI:* Sustained: -0.446kg/m^2^ (-0.88,-0.02); Non-sustained: 0.12 kg/m^2^ (-0.11, 0.35); ***P*=.049**; *METmax:* Sustained: 0.54 (0.29,0.78); Non-sustained: 0.42 (0.21, 0.62); *P*=.39; *SBP:* Sustained: -0.44mmHg (-4.35, 3.47); Non-sustained: 1.54mmHg (-1.12,4.23); *P*=.65; *DBP:* Sustained: 1.06mmHg (-1.48,3.61); Non-sustained: 1.70mmHg (0.18, 3.21); *P*=.64; *Triglycerides:* Sustained: -0.08mmol/l (-0.34,0.18); Non-sustained: -0.09mmol/l (0.20, 0.02); *P*=.55; *Total cholesterol:* Sustained: 0.28 mmol/l (0.06,0.49); Non-sustained: 0.27mmol/l (0.11, 0.42); *P*=.91 |
| Oh et al. (2018),^21^  South Korea  RCT  24 wk intervention duration; 24-wk follow-up | 422 adults w/ obesity & metabolic syndrome enrolled from 2 hospitals (CS)  Age: 48.6 (13.7)  Sex: 51% male  BMI: 29.4 (3.5)  Race: 100% Asian | I: Smart Care app, body comp. meter, pedometer to self-monitor body comp. & PA. Also, w/ feedback, education, exercise, meds, & nutrition phone consults.  C: Scale, pedometer, weight journal, info. on nutrition & exercise, anthropometry consults, & counseling. | User engagement: Times/wk weight, BMI, body fat, & waist circ. entered into app:  (1) Active (≥3x/wk)  (2) Less active (<3x/wk)  Main outcomes: BMI, body fat, weight, waist circumference, & lipid panel | *Weight change:* Active users: –3.18kg (0.29); Less-active users: –0.70kg (0.35); ***P*=.001**  *BMI:* Active: 28.77 kg/m^2^ (3.09); Less-active: 29.72 kg/m^2^ (3.11); ***P*=.03**  No stat. sig. diff in lipid panel improvement btw usage groups (actual data not available).  (Actual data not available for body fat & waist circumference). |
| Vehi et al. (2019),^36^  Spain  Retrospective study - 2 analyses  3 & 6 mo periods examined | Adults w/ T2DM logging into SDA ≥5 days/mo for ≥6 mos selected via CS.  *Analysis 1*, n=67  Age: 51.2 (4.07)  Sex: 75% male  BMI: no data  Race: no data  *Analysis 2,* n=444  Age: 52.0 (4.31)  Sex: 79% male  BMI: no data  Race: no data | SocialDiabetes (SDA) platform consists of app & desktop solution. Self- monitoring of BG, exercises, diet, & lifestyle; reminders; remote monitoring from healthcare professional; exercise coaching & charts; & meal planning. | User engagement:  *Analysis 1:* High engagement (group A) = logging in ≥15 x/mo for ≥6 mos & low engagement (group B) = logging in 5-10 x/mo for ≥6 mos.  *Analysis 2:* Group A = logging in ≥60 x/mo for ≥6 mos & group B = 5-10 x/mo for ≥6 mos.  Main outcomes:  *Analysis 1:* HbA1c *Analysis 2:* LBGI/HBGI | *Analysis 1:* mean BG reduction was 21% (***P*<.001**) & 30% (***P*<.001**) at 3 mos for groups A & B, respectively. Mean BG reduction was 27% (***P*<.001**) & 26% (***P*<.001**) at 6 mos for groups A & B, respectively. 6 mo reduction corresponds to a reduction of estimated HbA1c of approximately 2% for both groups A & B, respectively  *Analysis 2:* mean reduction in LBGI was 25% (***P*=.01**) & 19% (***P*=.03**) at 6 mos for groups A & B, respectively. Mean reduction in HBGI was 56% (***P*<.001**) & 44% (***P*<.001**) at 6 mos for groups A & B, respectively. No 3 mo results. |
| ‡ (95%CI:X,X)=(X,X); § mean(SD) unless otherwise specified; † Convenience sampling; Diagnosis (Dx); Hypertension (HTN); Diabetes (DM); Hemoglobin A1c (HbA1c); Blood glucose (BG); Primary Care Provider (PCP); Heart rate (HR); Systolic blood pressure (SBP); Diastolic blood pressure (DBP); High-density lipoprotein (HDL); Maximal metabolic equivalent value (METmax); Low/high blood glucose index (LBGI or HBGI). | | | | |
