# Supplementary material for: User Engagement With Smartphone Apps and Cardiovascular Disease Risk Factor Outcomes: Systematic Review
Source: JMIR Cardio. 2021 Feb 3;5(1):e18834. doi: 10.2196/18834 (PMC8411427; doi:10.2196/18834)
Supplement: Multimedia Appendix 3 [file cardio_v5i1e18834_app3.docx]

**Multimedia Appendices**

**Multimedia Appendix 3.** Quality assessment of studies included in this systematic review using the Joanna Briggs Checklist for analytical cross-sectional studies.

| Quality assessment criteria | Bennett et al [23] | Godino et al [17] | Johnston et al [19] | Kim et al [33] | Kim et al [34] | Lin et al [24] | Patel et al [25] | Patel et al [26] | Serrano et al [35] | Tanaka et al [27] | van Beurden et al [28] | Duncan et al [16] | Edney et al [30] | Garcia-Ortiz et al [29] | Mitchell et al [37] | Tong et al [38] | Wang et al [22] | Agarwal et al [32] | Bradway et al [31] | Hartin et al [18] | Koot et al [39] | Mattila et al [20] | Oh et al [21] | Vehi et al [36] | Total count of yes | Total count of no | Total count of unclear | Total count of N/A^a^ |
| --- | --- | --- | --- | --- | --- | --- | --- | --- | --- | --- | --- | --- | --- | --- | --- | --- | --- | --- | --- | --- | --- | --- | --- | --- | --- | --- | --- | --- |
| 1. Were the criteria for inclusion in the sample clearly defined? | Y^b^ | N^c^ | N | N | N | Y | Y | Y | N | N | Y | N | N | N | N | Y | N | Y | Y | Y | Y | N | N | N | 10 | 14 | 0 | 0 |
| 2. Were the study subjects and the setting described in detail? | Y | Y | N | N | N | Y | Y | Y | N | N | Y | Y | Y | Y | Y | N | Y | Y | Y | Y | Y | Y | Y | N | 17 | 7 | 0 | 0 |
| 3. Was the exposure measured in a valid and reliable way? | U^d^ | U | U | U | U | U | U | U | U | U | U | U | U | U | U | U | U | U | U | U | U | U | U | U | 0 | 0 | 24 | 0 |
| 4. Were objective, standard criteria used to measure the condition? | Y | Y | Y | N/A | N/A | Y | Y | Y | Y | Y | Y | N/A | N/A | N/A | N/A | Y | Y | Y | Y | Y | Y | Y | Y | N | 17 | 1 | 0 | 6 |
| 5. Were confounding factors identified? | N | N | N | Y | Y | Y | N | N | N | Y | N | Y | Y | N | Y | N | Y | N | N | N | Y | Y | N | N | 10 | 14 | 0 | 0 |
| 6. Were strategies to deal with confounding factors stated? | N/A | N/A | N/A | N/A | N/A | N | N/A | N/A | N/A | Y | N/A | Y | Y | N/A | Y | N/A | Y | N/A | N/A | N/A | Y | Y | N/A | N/A | 7 | 1 | 0 | 16 |
| 7. Were the outcomes measured in a valid and reliable way? | Y | Y | Y | N | N | Y | Y | Y | N | Y | Y | Y | Y | Y | Y | Y | Y | Y | Y | Y | Y | Y | Y | Y | 21 | 3 | 0 | 0 |
| 8. Was appropriate statistical analysis used? | U | U | U | Y | Y | U | U | U | U | Y | U | Y | Y | U | Y | U | Y | U | U | U | Y | Y | U | U | 9 | 0 | 15 | 0 |
| Total count of yes per study | 4 | 3 | 2 | 2 | 2 | 5 | 4 | 4 | 1 | 5 | 4 | 5 | 5 | 2 | 5 | 3 | 6 | 4 | 4 | 4 | 7 | 6 | 3 | 1 |  |  |  |  |
| ^a^N/A: not applicable; ^b^Y: yes; ^c^N: no; ^d^U: unclear. | | | | | | | | | | | | | | | | | | | | | | | | | | | | |

**Question One.** The studies most frequently received a score of “N” if they did not clearly state their inclusion and exclusion criteria or if they did not indicate a language requirement in their eligibility criteria.

**Question Two.** The studies most frequently received a score of “N” if they did not indicate a time period and place of enrollment as well as if they did not include at least one sociodemographic variable for their study sample beyond age and gender.

**Question Three.** Each study received a score of “U” because none of them assessed the entire emotional, cognitive, and behavioral experience of user engagement. In addition, despite being an objective measure, it is unclear how valid/reliable app analytics are for measuring the behavioral concept/process of user engagement.^11^

**Question Four.** The studies received a score of “Y” if they had a cardiovascular disease risk factor and/or condition in their eligibility criteria that was assessed objectively. They received a “N” if they had a cardiovascular disease risk factor and/or condition in their eligibility criteria that was not assessed objectively. They received a score of “N/A” if they did not have a cardiovascular disease risk factor and/or condition in their eligibility criteria.

**Question Five.** Studies most frequently received a score of “N” if they did not identify confounding factors related to user engagement and the outcome of interest.

**Question Six**. Studies that received a score of “Y’ in question 5, received a score of “N/A” for this question if the type of analysis didn’t allow for ways to deal with confounding factors, a score of “N” if they did not state strategies to deal with confounding factors, and a score of “Y” if they did state strategies to deal with confounding factors in regards to the analysis assessing the relationship between user engagement and the outcome of interest.

**Question Seven**. Studies received a score of “N” if the outcome being assessed in the analysis, looking at the relationship between user engagement and the outcome of interest, was not assessed in a valid/reliable way (ex through self-report).

**Question Eight.** Studies received a score of “U” if they received a score of “N” for question five or six because if they did not identify or state strategies to deal with confounding factors, for assessing the relationship between user engagement and the outcome of interest, then it would be unclear if the correct statistical approach was used.
